# Trypanosomes can initiate nuclear export co-transcriptionally

**DOI:** 10.1093/nar/gky1136

**Published:** 2018-11-12

**Authors:** Carina Goos, Mario Dejung, Ann M Wehman, Elisabeth M-Natus, Johannes Schmidt, Jack Sunter, Markus Engstler, Falk Butter, Susanne Kramer

**Affiliations:** 1 Department of Cell and Developmental Biology, Biocenter, University of Würzburg, Am Hubland, 97074 Würzburg, Germany; 2 Institute of Molecular Biology (IMB), Ackermannweg 4, 55128 Mainz, Germany; 3 Rudolf Virchow Center, University of Würzburg, Josef-Schneider-Strasse 2, 97080 Würzburg, Germany; 4 Department of Biological and Medical Sciences, Oxford Brookes University, Oxford OX3 0BP, UK

## Abstract

The nuclear envelope serves as important messenger RNA (mRNA) surveillance system. In yeast and human, several control systems act in parallel to prevent nuclear export of unprocessed mRNAs. Trypanosomes lack homologues to most of the involved proteins and their nuclear mRNA metabolism is non-conventional exemplified by polycistronic transcription and mRNA processing by trans-splicing. We here visualized nuclear export in trypanosomes by intra- and intermolecular multi-colour single molecule FISH. We found that, in striking contrast to other eukaryotes, the initiation of nuclear export requires neither the completion of transcription nor splicing. Nevertheless, we show that unspliced mRNAs are mostly prevented from reaching the nucleus-distant cytoplasm and instead accumulate at the nuclear periphery in cytoplasmic nuclear periphery granules (NPGs). Further characterization of NPGs by electron microscopy and proteomics revealed that the granules are located at the cytoplasmic site of the nuclear pores and contain most cytoplasmic RNA-binding proteins but none of the major translation initiation factors, consistent with a function in preventing faulty mRNAs from reaching translation. Our data indicate that trypanosomes regulate the completion of nuclear export, rather than the initiation. Nuclear export control remains poorly understood, in any organism, and the described way of control may not be restricted to trypanosomes.

## INTRODUCTION

Eukaryotic messenger RNA (mRNAs) require several nuclear maturation steps. The nuclear envelope serves as the main mRNA surveillance system and ensures that only mature mRNAs reach the cytoplasm. During transcription and processing, each mRNA molecule successively associates with a set of proteins that either direct it to degradation or facilitate export (reviewed in (1)). The TRAMP complex marks faulty mRNAs for degradation by the nuclear RNA exosome (2). Successful processing decorates the mRNA with a set of shuttling adaptor proteins, often serine arginine (SR) rich proteins, which then recruit the nuclear export receptor complex Mex67/Mtr2 and thus mark the mRNA as export competent. The export-competence of each messenger ribonucleoprotein (mRNP) is further controlled at the entrance site of the nuclear pore (the nuclear basket). Transcription and nuclear export are tightly connected by the transcription/export (TREX) complex. Even though several factors were identified that play a role in mRNA quality control, the underlying mechanism still remains largely elusive. In particular, it remains unclear whether an mRNP is actively selected for nuclear export if it is intact, or actively retained if it is faulty, or, whether both models are involved (3). Furthermore, it remains largely unclear how faulty mRNAs are distinguished from intact ones, although post-translational modifications of SR proteins may play a role (4).

Trypanosomes are unique in that their entire genome is transcribed into long polycistrons, each containing tens to hundreds of genes (5–7). Promoters are largely absent and transcription start and stop sites are defined by histone variants (8). Individual mRNA molecules are produced by trans-splicing; this process adds the capped 39-nt long exon of the spliced leader RNA to the 5′ end of each mRNA, and is coupled with polyadenylation of the upstream gene (9). With two exceptions, trypanosome genes have no introns (5,10–12). Trypanosomes have a TRAMP-like complex (13) and a nuclear RNA exosome (14), which degrades unspliced RNA (15) as well as a functional homologue to the Mex67/Mtr2 complex (16,17). Surprisingly, trypanosomes also use the export factors NMD3 and XPO1, (which are mainly involved in the export of ribosomal RNAs) in addition or in parallel to the Mex67/Mtr2 pathway for mRNA export (18). Apart from these proteins, the parasites lack homologues to any of the yeast proteins that act as export adaptors or to the mRNA control factors at the nuclear basket (19,20); whether trypanosomes have a TREX complex remains controversial (21). Moreover, trypanosomes have a highly unusual nuclear pore structure that largely lacks the usual asymmetry of nuclear pore components seen in opisthokonts (20,22).

As in other eukaryotes, nuclear export control is poorly understood in trypanosomes. By fractionation methods, it was observed that unspliced (e.g. polycistronic) mRNAs can reach the cytoplasm (23,24), indicating that any control system is not tight. However, some control system must be present, since the concentration of unspliced *tubulin* mRNA was found to be 8-fold higher in the nucleus than in the cytoplasm (23) and polycistronic transcripts accumulate upon RNA interference (RNAi) depletion of the nuclear RNA exosome (15). We have previously investigated nuclear export in trypanosomes by inhibiting trans-splicing. This caused the formation of a novel cytoplasmic ribonucleoprotein granule type localized at the periphery of the nucleus, termed nuclear periphery granules (NPGs) (23). Several cytoplasmic RNA-binding proteins were shown to localize to the granules, for example the DEAD box RNA helicase DHH1, the Lsm domain protein SCD6 and Poly(A) binding protein 2 (PABP2), while proteins involved in earlier nuclear mRNA processing steps were absent (23). The function of NPGs remains unknown, but the granule’s dependency on active transcription but not on translation suggests that they may act as a cytoplasmic mRNA quality control compartment.

Here, we used intra-molecular multi-colour single molecule fluorescence *in situ* hybridization (smFISH) to examine nuclear export of intact and unspliced transcripts in more detail. We found that nuclear export can start co-transcriptionally and thus, in the absence of splicing. However, unspliced transcripts are largely prevented from reaching the peripheral cytoplasm and instead appear stuck in nuclear pores and accumulate in NPGs. A closer analysis of NPGs by electron microscopy and proteomics revealed that the granules are localized at the cytoplasmic side of nuclear pores and contain all proteins also present in cytoplasmic stress granules. Thus, NPGs are the result of an interrupted nuclear export process and contain part of unspliced mRNAs (decorated with the standard set of RNA-binding proteins) trapped in the pore, indicating a nuclear export control mechanism that acts during the export of faulty transcripts across the nuclear pore rather than in the nucleoplasm.

## MATERIALS AND METHODS

### Trypanosomes

*Trypanosoma brucei* Lister 427 procyclic wild-type cells were used throughout, as well as variants expressing one or two fluorescently labelled fusion proteins from the endogenous locus. These cell lines had no growth phenotypes and the functionality of the key fusion proteins eYFP-DHH1 and SCD6-eYFP had been shown by deleting the wild-type allele (25,26). SCD6 overexpression was done as described (26) and causes a growth phenotype, albeit not within the 24 h of induction that were used for the experiments here (26). RNAi depletion of MEX67 was done in cells expressing PSPR2.1 (Tet repressor) and containing a plasmid for tetracycline inducible expression of hairpin Mex67 RNA based on p3666 (27). All experiments were performed with logarithmically growing trypanosomes at a cell density of <1 × 10^7^ cells/ml. The generation of transgenic trypanosomes was done using standard procedures (28). For inhibition of trans-splicing, cells were incubated with 2 μg/ml sinefungin for 60 min, with the exception of the purification of the NPGs that used 0.2 μg/ml. Alternatively, trans-splicing was inhibited with a morpholino antisense to Tb U2 snRNA as previously described (23), controlled with a standard control morpholino provided by GeneTools (5′CCTCTTACCTCAGTTACAATTTATA 3′). Transcription was inhibited with actinomycin D (10 μg/ml) for 60 min. For starvation, cells were washed once in one volume phosphate buffered saline (PBS) and incubated in PBS for 120 min.

### Western blots and antibodies

Western blots were performed according to standard protocols. The following antibodies were used: anti *T. brucei* SCD6 (25), anti *T. brucei* DHH1 (25), anti *T. brucei* histone H3 (29), BB2 monoclonal antibody and anti-BiP. For immunofluorescence and immunogold EM experiments, SCD6 and DHH1 antibodies were affinity purified with the aid of recombinant SCD6 or DHH1 proteins, using standard procedures. Proteins were detected and quantified by the Odyssey Infrared Imaging System (LI-COR Biosciences, Lincoln, NE). Background method for quantification: the average of a 3-pixel width line at the top and bottom of each band was subtracted from each pixel.

### Purification of NPGs

Initial test experiments were done with a cell line expressing the granule marker eYFP-DHH1 from the endogenous locus (25) (Figure 6C and D). Later, a cell line co-expressing mChFP-DHH1 (25) and PABP2–4Ty1-eYFP (mother plasmid SK141 (30)) from the endogenous loci was used, to enable the detection of PABP2 on a western blot (Figure 6E). The purification protocol was based on the purification of trypanosome nuclei described in (31), modified as described in (32).

### Mass spectrometry

About 600 μl methanol, 150 μl chloroform and 450 μl water were added stepwise (with vigorous vortexing after each step) to 200 μl (10%) of the pellet fraction. After centrifugation (5 min, 20 000 g), the upper, aqueous phase was discarded, and another 650 μl methanol was added and mixed by inversion. Proteins were pelleted by centrifugation (5 min, max. speed), resuspended in 100 μl 4× NuPAGE LDS sample buffer (Thermo Fisher Scientific) with 100 mM dithiothreitol (DTT) and incubated at 70°C for 10 min. Afterwards the samples were sonicated with the Bioruptor^®^ Plus sonication device (Diagenode, Belgium) (settings: high, 10 cycles, 30 s ON /30 s OFF).

The samples were in-gel digested and MS measurement performed as previously described (33) with the following adaptations: the measurement time per sample was extended to 240 min. The triplicates were analysed with MaxQuant version 1.5.0.25 (34) with standard settings except LFQ quantitation and match between runs was activated. The trypanosome protein database TREU927 version 8.0 (11 567 entries) was downloaded from www.tritrypdb.org (35). Further analysis was conducted in the Perseus environment (36) with filtering for proteins only identified by site, reverse entries, potential contaminants and quantitation values in at least 2 of the 3 replicates. Prior to imputation of missing LFQ values with a normal distribution (width 0.3, downshift 1.8), the LFQ values were log2 transformed. Significant enriched proteins were determined using a Welch *t*-test with 250 randomizations at a false discovery rate (FDR) = 0.05 and *S*_0_ = 0.5. The data was exported (Supplementary Table S1C). The volcano plot (Figure 6F) was generated with the R ggplot2 package.

### Localization of ESTN proteins

The localization of ESTN (enriched in sinefungin treated nuclei) proteins was determined by expressing eYFP fusion proteins (C-terminal tagging) in a cell line co-expressing the NPG granule marker mChFP-DHH1 from the endogenous locus (p2845) (25). The fusion proteins were made either according to (37) (17/118 proteins, using SK141, which adds a double tag out of 4 ty1 and eYFP) or (38) (101/118 proteins, using pPOTv4). The 17 proteins tagged according to (37) were Tb927.9.11050, Tb927.9.14120, Tb927.9.8520, Tb927.11.13970, Tb927.11.16610, Tb927.11.4900, Tb927.10.12980, Tb927.9.5460, Tb927.11.6600, Tb927.11.2250, Tb927.7.3040, Tb927.11.1890, Tb927.11.6720, Tb927.4.3350, Tb927.5.1490.

Tb927.10.1510 and Tb927.3.1920; the correct size of these fusion proteins was controlled by western blot probed for the Ty1 epitope tag by the BB2 antibody (data not shown). Only proteins that unequivocally co-localized with mChFP-DHH1 to NPGs at sinefungin treatment were considered true NPG proteins. Proteins with an expression level too low to be certain were classified as ‘not determined’.

### Fluorescence microscopy and image analysis

Images were recorded either with a custom-built TILL Photonics iMIC microscope (Z-stacks with 100 images and 100 nm spacing) or with an inverted fully automated DMI6000 wide-field microscope from Leica Microsystems, (Mannheim, Germany) (Z-stacks with 50 images and 140 nm spacing), both equipped with 100× oil objectives (NA 1.4). All stacks were deconvolved using Huygens Essential software (SVI, Hilversum, The Netherlands). Unless stated otherwise, images are shown and analysed as projections of deconvolved Z-stacks. Super resolution images were done on a Zeiss Elyra S.1 SIM - Super Resolution Microscope with a 63× oil objective and an sCMOS-Camera (PCO Edge 5.5) (21 stacks with 124 nm spacing); a single plane image is shown.

To determine the nuclear border in cells expressing NUP96-eYFP, the focus plane of a Z-stack (50 stacks at 140 nm) was automatically determined with ImageJ on the DAPI (4′,6-diamidino-2-phenylindole) stack (Plugin ‘Find Focused Slices’ 100% maximum variance, verbose mode); the same plane was used for the NUP96 stack. For analysis, a merged image was built of the single NUP96 plane and the DAPI Z-stack projection (sum slices).

### smRNA FISH (Affymetrix)

smRNA FISH was done as previously described (39). The sequences used to design the Affymetrix FISH probes are in Supplementary Table S2. For combination with immunofluorescence, slides were left in PBS/4°C over night, followed by immunofluorescence the next day: slides were blocked in 2% bovine serum albumin (BSA)/PBS for 1 h, incubated with affinity purified anti-DHH1 (1:500) in 2%BSA/PBS for 1 h, washed four times in PBS (5 min each wash), incubated with secondary antibody (1:1000 in 2%BSA/PBS; Alexa 488 or Alexa 594 goat anti rabbit), washed four times in PBS (5 min each wash), stained with DAPI (1 min, 1.25 μg/ml) and embedded in Prolong Gold antifade.

### Electron microscopy

The high pressure freezing protocol is adapted from (40–42). Fifteen-mililiter PCF (procyclic form) culture at 5 × 10^6^ cells/ml were centrifuged (750 g, 3 min, RT). All but 2 ml medium were removed and 2 ml heat-inactivated fetal calf serum was added as a cryoprotectant. Cells were centrifuged again (750 g, 3 min, RT) and the pellet was transferred to a polymerase chain reaction (PCR) tube and further compacted (5 s, minifuge). A drop of the final pellet was transferred to the freezing container (specimen carriers type A, 100 μm, covered with specimen type B, 0 μm, Leica Microsystems). High pressure freezing was done in an EM HPM100 (Leica Microsystems) at a freezing speed >20 000 Ks^−1^ and a pressure >2100 bar. The samples were stored in liquid nitrogen until freeze substitution in an EM AFS2 freeze substitution system (Leica Microsystems).

For embedding in epon (to visualize nuclear pore complexes), samples were incubated in 0.1% (w/v) tannic acid and 0.5% (v/v) glutaraldehyde in anhydrous acetone at −90°C for 96 h (with one change in solution after 24 h), washed four times for 1 h with anhydrous acetone at −90°C and fixed in 2% OsO_4_ (w/v) in anhydrous acetone at −90°C for 28 h. Then the temperature was gradually raised to −20°C within 14 h, kept at −20°C for 16 h and gradually raised to 4°C within 4 h. Afterwards samples were immediately washed with anhydrous acetone at 4°C four times at 0.5 h intervals, followed by gradually increasing the temperature to 20°C within 1 h. Subsequently, samples were transferred for embedding into increasing concentrations of Epon (50% Epon in acetone for 3 h at room temperature, 90% Epon in acetone overnight at 4°C, followed by two times 100% Epon at room temperature for 2 h, all solutions were freshly prepared). Epon infiltrated samples were polymerized for 72 h at 60°C.

For immunogold-labelling, embedding was done with LR-white. Samples were incubated in 0.1% KMnO_4_ in anhydrous acetone at −90°C for 65 h, the solution was exchanged once with fresh 0.1% KMnO_4_ in anhydrous acetone and temperature was ramped to −45°C (4°C/h). Samples were washed four times with anhydrous acetone over 3 h, one time with 1/3 ethanol in acetone for 30 min, one time with 2/3 ethanol in acetone for 30 min and finally two times with 100% ethanol for 30 min each. Afterwards the temperature was gradually raised to 4°C (4°C/h) and the samples were washed twice with 100% ethanol for 30 min each. Subsequently, samples were transferred for embedding into freshly prepared solutions of LR-White (50% LR-White in ethanol for 3 h at 4°C, 50% LR-White in ethanol over night at 4°C, followed by three times 100% LR-White for 1 h, 3 h and over night at 4°C). LR-White infiltrated samples were polymerized for 72 h at 52°C.

Sixty-nanometre serial sections were cut with an ‘ultra Jumbo Diamond Knife’ (Diatome AG) and placed on either pioloform coated slotted copper grids or 100 mesh nickel grids depending on the subsequent analysis.

For immunogold labelling, sections on nickel grids were rehydrated with PBS (15 min), blocked with 1% BSA/ 0.1% Tween20 (25 min) and stained with an affinity purified *T. brucei* SCD6 antibody (2 h, RT, 1:500 in PBS) followed by four washing steps with 0.1% BSA/0.1% Tween20 (each 10 min). Sections were incubated with a secondary antibody, 12 nm gold conjugated goat anti rabbit IgG (Jackson Immuno research (111–205-144)) (2 h, RT, 1:10 in 0.1% BSA/ 0.1% Tween20). Finally, sections were washed twice in 0.1% BSA/ 0.1% Tween20, twice in PBS, once in 1.25% glutaraldehyde in PBS and three times in ddH_2_O (each 15 min).

For staining and contrasting, Epon embedded sections were incubated in 2% aqueous uranyl acetate for 10 min followed by incubation in Reynolds lead citrate for 5 min. LR White embedded sections were incubated in 2% aqueous uranyl acetate for 5 min followed by incubation in Reynolds lead citrate for 1.5 min. A 200 kV JEM-2100 (JEOL) transmission electron microscope equipped with a TemCam F416 4k x 4k camera (Tietz Video and Imaging Processing Systems) was used for imaging.

## RESULTS

### Defining the nuclear border by DAPI staining

**Figure 1.**
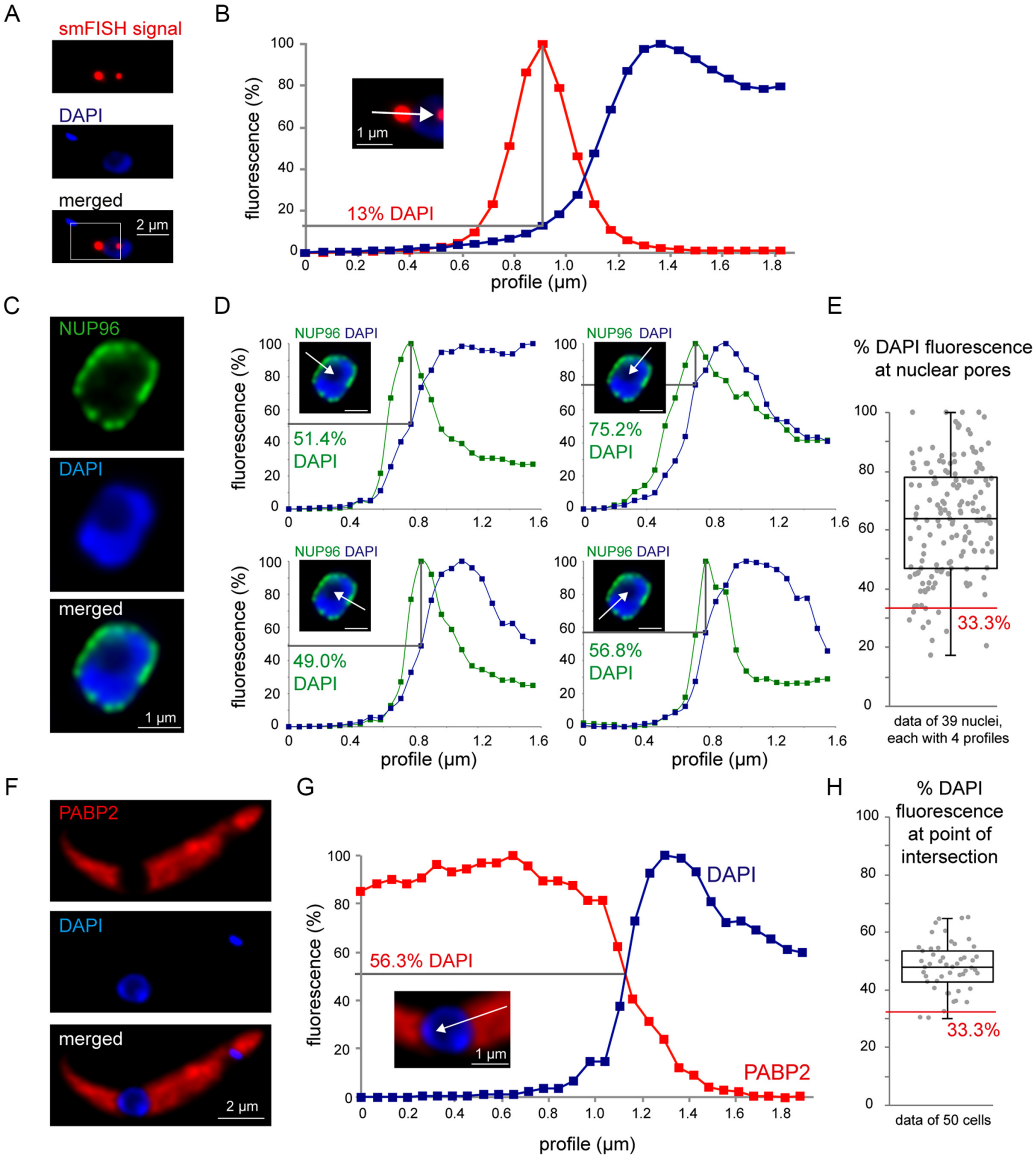
Defining the nuclear border by DAPI staining. (**A** and **B**) smFISH signals at the nuclear border are described as position on a DAPI profile (white arrow in B) that ranges from the cytoplasm (0% DAPI) via a peak (100% DAPI) to the nucleolus (<100% DAPI). An example image is shown (smFISH with the red fluorescent 5′ probe of Tb927.1.1740) (A), with the smFISH signal localized at 13% DAPI (B). (**C–E**) Cells expressing the nuclear pore protein NUP96-eYFP from the endogenous locus were fixed and stained with DAPI. (C) One image of a nucleus is shown. (D) Four different profiles of the same nucleus are shown with the maximal fluorescence of NUP96 across the DAPI profile indicated as %DAPI; the scale bar is 1 μm. (E) The position of NUP96 across the DAPI profile was quantified for 39 nuclei, each with four profiles. The red line marks 33.3% DAPI fluorescence. (**F–H**) Cells expressing a fluorescent cytoplasmic protein (PABP2-mChFP) were fixed and stained with DAPI. The red fluorescence and the DAPI fluorescence were measured along a line (profile) ranging from the cytoplasm to the nucleolus and both were calibrated to a 0–100% scale. The point of intersection was calculated. An example cell is shown in panel F; the profile is shown in panel G. The full dataset of 50 cells is shown in panel H.

For the analysis of nuclear mRNA export by smFISH it is essential to describe the localization of an smFISH signal in correlation to the nuclear membrane. Throughout this work, we describe an smFISH signal as position on a linear DAPI fluorescence profile (% DAPI), ranging from the cytoplasm (0% DAPI fluorescence) via a peak (100% DAPI fluorescence) to the nucleolus (<100% DAPI fluorescence). An example of such quantification is shown in Figure 1A and B: this mRNA molecule is localized at 13% DAPI fluorescence.

Two control experiments were done to correlate the DAPI fluorescence to the nuclear border. First, nuclear pores were visualized by expressing a C-terminal eYFP fusion of the nuclear pore protein *Tb*NUP96 from the endogenous locus (23). Nuclear pores peaked at 63±19% DAPI (*N* = 156), with 6.4% of pores at <33% DAPI fluorescence (Figure 1C–E). Second, the cytoplasm was labelled by expressing a C-terminal mCherry fusion of the cytoplasmic protein PABP2 from the endogenous locus (43). The cytoplasmic fluorescence intercalated with the DAPI profile at 48%±9 (*N* = 50), with only 4% at >33% DAPI fluorescence (Figure 1F–H). From these control experiments, we conclude that the nuclear border is in average between 50 and 60% DAPI fluorescence. The variance between the cells means that the DAPI profile cannot be used to define the nuclear border in individual cells, however, the likelihood that any smFISH signal at >33% DAPI fluorescence is cytoplasmic is high.

### Intramolecular three-colour smFISH across the nuclear border

The 22 197-nt long mRNA Tb927.1.1740, encoding the putative microtubule-associated protein FUTSCH, has been previously used for the detection of mRNA transcription and decay intermediates by dual colour smFISH: the 1100 nt at the 5′ end were labelled with a red fluorescent probe, the 1100 nt at the 3′ end with a green fluorescent probe. With this system, red foci represent mRNAs in transcription or 3′-5′ decay, green foci mRNAs in 5′-3′ decay and yellow foci (red+green) are intact mRNA molecules (39). In average, there are 4.3±3 *FUTSCH* mRNA molecules per cell; 21% are yellow, 31% are red and 48% are green. Red foci are mainly in the nucleus or at the nuclear periphery and largely insensitive to inhibition of transcription by actinomycin D, indicating that these are transcription intermediates. Green foci are mainly in the cytoplasm and sensitive to actinomycin D treatment, consistent with these being 5′-3′decay intermediates. Yellow foci are distributed in both nucleus and cytoplasm. There has been some debate as to whether *FUTSCH* is a ‘true’ protein encoding mRNA. However, cycloheximide treatment causes a 2.2-fold increase in intact mRNA molecules on expense of 5′-3′decay intermediates. The drug blocks translational elongation and prevents the release of mRNAs from polysomes, and the increase in intact *FUTSCH* mRNA molecules at cycloheximide treatment is thus strong evidence for its polysomal localization, and thus translation (39). No increase in intact *FUTSCH* mRNA is observed when translation is inhibited with puromycin, a translational inhibitor that causes polysome dissociation, indicating that stabilization of *FUTSCH* by cycloheximide is not caused by a secondary effect of the translational inhibition per se (39). Furthermore, both 5′ splice sites and polyadenylation sites were mapped by RNA sequencing (44) and we have confirmed the presence of a spliced leader by RT-PCR (Supplementary Figure S14).

**Figure 2.**
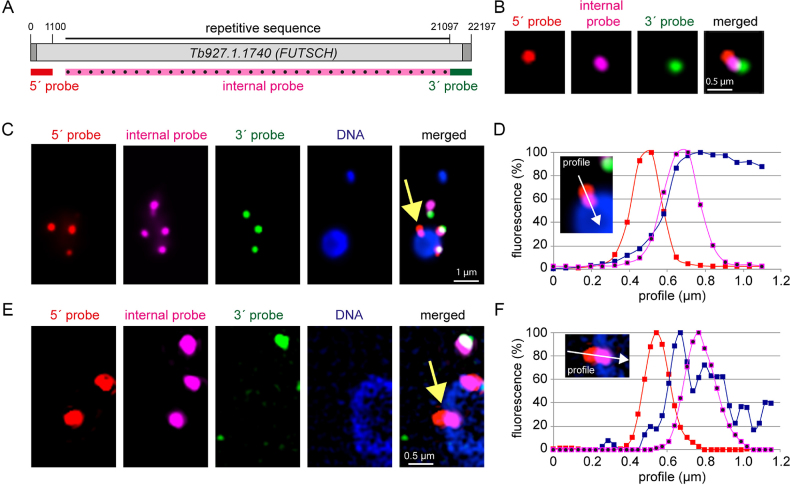
Visualization of single mRNA molecules crossing the nuclear border. (**A**) To-scale schematics of the endogenous reporter gene Tb927.1.1740 (*FUTSCH*) used for three colour intra-molecular smFISH FISH. The positions of the three probes are indicated. The internal probe uses an infrared dye that is shown in pink false colour throughout. (**B**) Example of a full-length mRNA molecule in an extended form (Z-projection). Note that most mRNA molecules have a more complex secondary structure that does not allow such clear separation of the three colours. (**C–F**) Example of a red-pink stained mRNA in nuclear export (yellow arrow) imaged with epifluorescence microscopy (C) or structured illumination microscopy (E). The fluorescence profiles (white arrow, calibrated to a 0–100% scale) are shown for each probe together with DAPI (D and F).

For visualization of nuclear export, the system was extended such as most of the remaining sequence of the mRNA was probed with an infrared probe (false-coloured in pink throughout) (Figure 2A). An example of an intact mRNA molecule (probed in three colours) is shown in Figure 2B; note that mRNA folding usually causes the three differently coloured ‘foci’ to be close to each other, rarely extending beyond 0.5 μm and never reaching the theoretical distance of 7.5 μm (39). Examples of whole cell images are shown in Supplementary Figure S1. Next, we used the system to specifically look at transcripts at the nuclear border: we found many cells with a red signal likely outside the nucleus (at <33% DAPI fluorescence) and a pink signal inside the nucleus or at the nuclear border, that, surprisingly, had no green signal nearby (Figure 2C and D). This was confirmed in higher resolution by structured illumination microscopy (Figure 2E and F).

### Trypanosome can start nuclear export of mRNAs during their transcription

**Figure 3.**
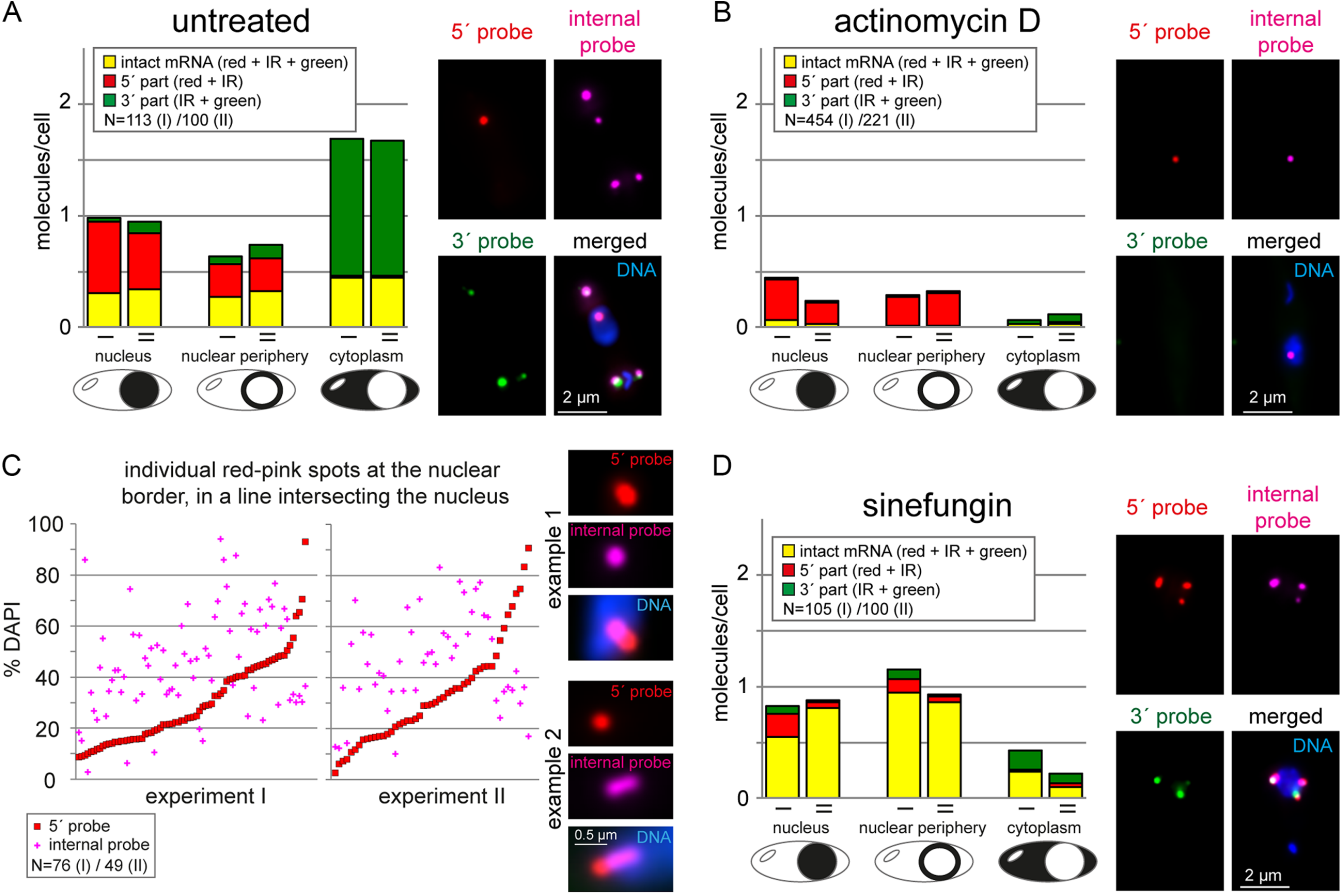
Nuclear export requires neither completion of transcription, nor of splicing. Three colour intramolecular smFISH of Tb927.1.1740 (compare Fig 2A). Data of two biological replicates (I and II) are shown for all experiments. (**A**) The distinct mRNA molecules (intact = stained in red, infrared (IR) and green; 5′ part = stained in red and IR; 3′ part = stained in IR and green) were classified according to their localization (nucleus, nuclear periphery, cytoplasm). Any molecule that was localized at the nuclear border and had at least one of its FISH signals between 66.6 and 10% DAPI fluorescence was classified as ‘nuclear periphery’. One example image is shown. (**B**) Cells were treated with actinomycin D for 60 min and analysed as in A. (**C**) Cells treated with actinomycin D for 60 min were searched for red-pink spots at the nuclear border that were in a line intersecting with the nucleus. The % DAPI fluorescence was determined for both the red and the pink foci and is plotted for each individual spot. For better visualization, the data were sorted according to the % DAPI fluorescence at the red spot. Pink signals above the red line thus correspond to red-pink foci that have the red signal closer to the cytoplasm than the pink signal and thus have the expected orientation of mRNA export. Two example nuclei used for this quantification are shown. The first is a typical example; the second is a rare example with an elongated mRNA molecule, likely reflecting a transcription site distant to the site of export. (**D**) Cells were treated with sinefungin for 60 min and analysed as in A.

Quantification revealed that ^1^/_3_ of all red-pink foci pairs are at the nuclear periphery (with at least one of the two signals between 10 and 66% DAPI) (Figure 3A). Of these red-pink foci pairs at the nuclear periphery, ^2^/_3_ had at least one of the colours (mostly red) likely in the cytoplasm. It should be noted that this estimate is conservative, as the smFISH procedure results in cells too thin for a meaningful 3D analysis. Thus, only mRNAs at the ‘edge’ of the nucleus in a 2D image could be analysed and red-pink spots on top or bottom of the nucleus are wrongly classified as nucleus-localized.

Red-pink foci can be either mRNAs in transcription, or mRNAs in 3′-5′ decay. To distinguish between these two possibilities, we analysed cells treated with the transcriptional inhibitor actinomycin D, a drug that specifically blocks transcription elongation by intercalation into G-C rich stretches of the DNA (45–49). If the red-pink foci were transcription intermediates, they would remain anchored at the site of transcription for a certain period, while intact mRNA molecules and all decay intermediates would continue decay. In fact, after 60 min of actinomycin D treatment, the number of intact and 5′-3′ decay intermediates was massively reduced to 7%, while the number of red-pink spots was only slightly reduced to 65% and now comprised >^3^/_4_ of all molecules (Figure 3B). These data show that most or all of the red-pink foci are mRNAs in transcription and not in 3′-5′ decay.

We used the actinomycin D treated cells for a quantitative analysis of the orientation of the mRNA molecules during nuclear export: for 81% of all mRNA molecules that had the red and pink spot orientated in a line intersecting the nucleus, the red spot was at a lower DAPI fluorescence and thus further towards the nuclear periphery than the pink spot, consistent with the 5′ end being exported first (Figure 3C). The remaining 19% of molecules may be exported 3′-5′, but it is more likely that they are artefacts of the method: data were analysed as Z-stack projections and if an mRNA’s 5′ end loops ‘backwards’ after nuclear export, it will be wrongly classified as 3′ to 5′ export, as we cannot distinguish FISH signals within the nucleus from FISH signals on top of the nucleus.

Taken together, the data strongly suggest that trypanosomes can export mRNAs co-transcriptionally. These observations are not restricted to the one reporter mRNA, as we have also analysed another long RNA (Tb927.04.310) by 2-colour smRNA FISH co-staining the 5′ end and the 3′ end (39), with very similar results (Supplementary Figure S2).

### Trypanosome mRNAs can start nuclear export when splicing is inhibited

Polycistronically transcribed trypanosome mRNAs are processed to monocistronic mRNAs by trans-splicing coupled to polyadenylation of the upstream gene. If nuclear export can start co-transcriptionally, a consequence would be that the completion of trans-splicing and the presence of a poly(A) tail is not required for the start of nuclear export. To investigate this, we first did a control experiment and blocked mRNA export by RNAi depletion of the well-characterized and essential nuclear export factor MEX67 (16,17). As expected, we observed an increase in intact mRNAs in the nucleus, which was ∼3.5- fold after 24 h RNAi induction (Supplementary Figure S3). There was no obvious change in the number of mRNAs at the nuclear periphery (Supplementary Figure S3). We also observed an unexpected increase in intact mRNA molecules in the cytoplasm; the reason for this remains unknown and was not further investigated in this study (Supplementary Figure S3).

If trans-splicing is required for nuclear export, inhibition of trans-splicing should cause a similar accumulation of intact mRNAs inside the nucleus as inhibition of nuclear export. When we blocked trans-splicing with the splicing inhibitor sinefungin (50), we did observe a small increase in intact mRNAs in the nucleus of ∼2-fold. However, surprisingly, we also observed a 3.1-fold increase in full-length mRNAs at the nuclear periphery (Figure 3D) and most of these mRNAs (^2^/_3_) had at least one foci at <33% DAPI fluorescence and therefore most likely in the cytoplasm. These data strongly suggest that mRNAs can start nuclear export in the absence of splicing.

### Unspliced mRNAs accumulate at the cytoplasmic side of the nuclear envelope

**Figure 4.**
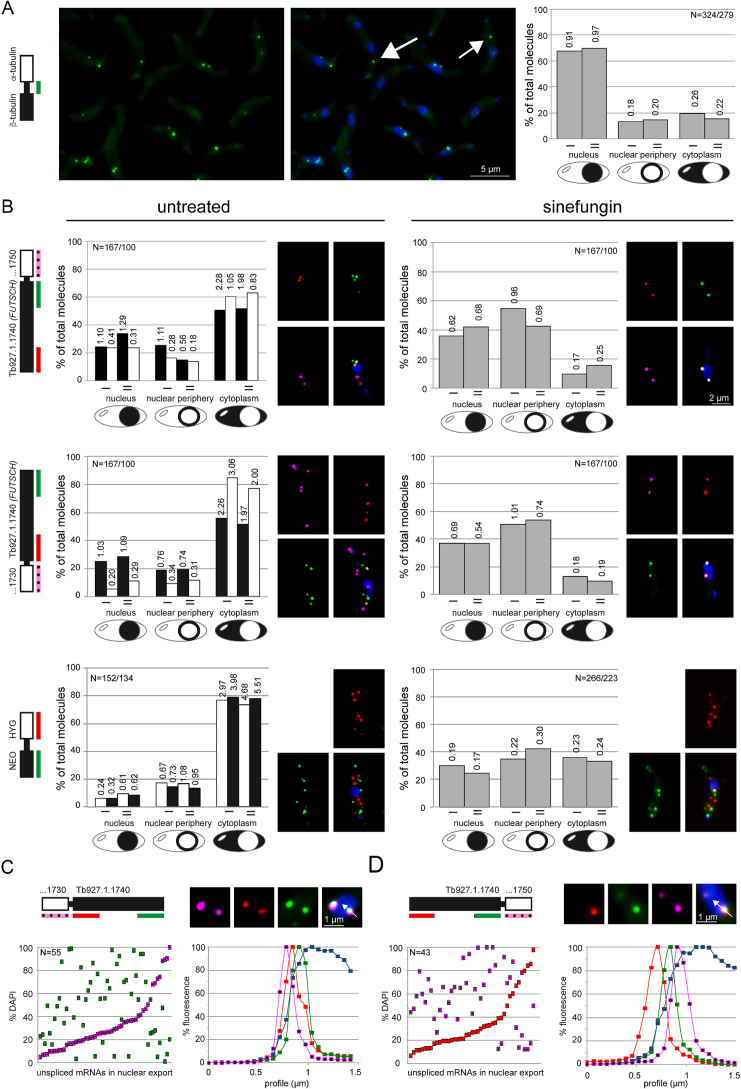
Unspliced mRNAs are enriched at the nuclear periphery. (**A**) Unspliced tubulin mRNAs were detected by a green smFISH probe antisense to the intergenic region between the adjacent β- and α-tubulin genes. One representative Z-projection image is shown (left) and as overlay with DAPI (middle). The localization of the unspliced tubulin mRNAs was determined in two independent experiments (I, II) (right). The numbers indicate average transcript numbers per cell and localization. (**B**) mRNAs of two adjacent genes were probed by two- or three- colour smFISH as indicated on the schematics on the left. The localization of individual transcripts (white and black bars) was quantified from untreated cells; for the two large mRNAs probed in two colours, the numbers of intact molecules, transcription and decay intermediates were added. The localization of unspliced transcripts (including all colour combinations across the gene border) was quantified after 60 min sinefungin treatment (grey bars). Data of two independent experiments (I, II) are shown with transcripts numbers per cell and localization indicated. Example images are shown. (**C** and **D**) Cells treated with sinefungin for 60 min were searched for pink-green (C) or red-pink (D) spots at the nuclear border that were in a line intersecting with the nucleus. The DAPI fluorescence was determined for both spots and is plotted. For better visualization, the data were sorted according to the DAPI fluorescence of the pink (C) or red (D) spots. An image of one example nucleus with fluorescence profile is shown for each mRNA. Representative data from one out of two independent experiments are shown.

Data from nuclear-cytoplasmic fractionation experiments suggest that in trypanosomes unspliced mRNAs can enter the cytoplasm (23,24). Consistently, probing the intergenic region between the adjacent β- and α-tubulin genes by smFISH confirms the presence of dicistronic tubulin mRNAs in the cytoplasm (Figure 4A). However, the majority (^2^/_3_) of tubulin dicistrons still remains in the nucleus (Figure 4A), while co-probing for two different mature mRNAs with open-reading frame probes shows that these are mainly cytoplasmic (Supplementary Figure S4), consistent with results from FISH experiments probing for global mRNAs (for example (23,51)). This argues that trypanosomes do have a control system to prevent the export of the majority of unspliced mRNAs to the cytoplasm.

To further investigate the localization of unspliced transcripts, we inhibited splicing with sinefungin and analysed three unspliced transcripts, each by probing two adjacent genes with smFISH (Figure 4B). First, the reporter mRNA Tb927.1.1740 (*FUTSCH*) described above was probed in red (5′ end) and green (3′ end) together with its downstream gene Tb927.1.1750 in infrared. Second, the same reporter mRNA was probed in red (5′ end) and green (3′ end) together with its upstream gene Tb927.1.1730 in infrared. Third, a plasmid was inserted to replace the endogenous *DBP1* gene by a neomycin resistance gene (NEO) and a hygromycin resistance gene (HYG); these genes were separated by the UTRs and intergenic region present between β- and α-*tubulin* (Supplementary Figure S5). This NEO-HYG transcript was chosen because the NEO and HYG mRNAs have average sizes, in contrast to the large size of the Tb927.1.1740 mRNA. It was possible to use this transgenic transcript rather than endogenous mRNAs as it is well established that *T. brucei* mRNA metabolism is mostly regulated by the UTRs rather than the ORF. *Tubulin* was not included to the analysis, because the mature *tubulin* mRNA is too abundant for quantification by smFISH and the data from an intergenic probe (as in Figure 4A) are difficult to analyse when trans-splicing is inhibited: the β and α *tubulin* genes are arranged in multiple tandems, and inhibition of trans-splicing causes the formation of a range of differently-sized polycistrons with larger *tubulin* polycistrons usually found in the nucleus ((23) and Supplementary Figure S6), consistent with nuclear retention being dependent on the number of splice-sites.

The localization of the individual mRNA molecules to either the nucleus the nuclear periphery or the cytoplasm was first analysed in untreated cells (Figure 4B, left panel). For all six individual mRNAs, the majority of molecules localized to the cytoplasm; the minor differences between the mRNAs likely result from differences in mRNA size and half-life, which influence the time of a molecule in transcription, nuclear export and decay. The percentage of unspliced mRNAs was with 1.8±0.4% very small (not shown).

Then, we inhibited splicing by sinefungin and monitored the localization of the unspliced transcripts. In contrast to the mature mRNAs, all three unspliced transcripts were enriched at the nuclear periphery and within the nucleus. The fraction that reached the cytoplasm outside of the nuclear periphery was at least 2-fold reduced in comparison to the spliced form. We also monitored the orientation for the fraction of the two large unspliced transcripts that had the 5′ end and the 3′ end clearly separated across the nuclear border, by determining the respective position on the DAPI profile. The majority of the unspliced transcripts (>70%) had the 5′ end at a lower DAPI fluorescence than the 3′ end, consistent with the correct 5′-3′ orientation of nuclear export (Figure 4C and D).

Sinefungin is an inhibitor of S-adenosyl methionine dependent methylations and inhibits trans-splicing by preventing the methylation of the SL RNA cap (50). To exclude that the observed changes in RNA localization were caused by unspecific effects of sinefungin, we also inhibited trans-splicing with a morpholino antisense to the U2 snRNA, controlled with an unspecific morpholino (23,52). We obtained similar results (Supplementary Figure S7), indicating that the accumulation of unspliced transcripts at the nuclear periphery is specific to the inhibition of splicing.

Taken together, the data indicate that unspliced transcripts accumulate at the cytoplasmic side of the nuclear membrane and in transport across the nuclear membrane.

### Unspliced mRNAs co-localize with nuclear periphery granules at the nuclear pores

**Figure 5.**
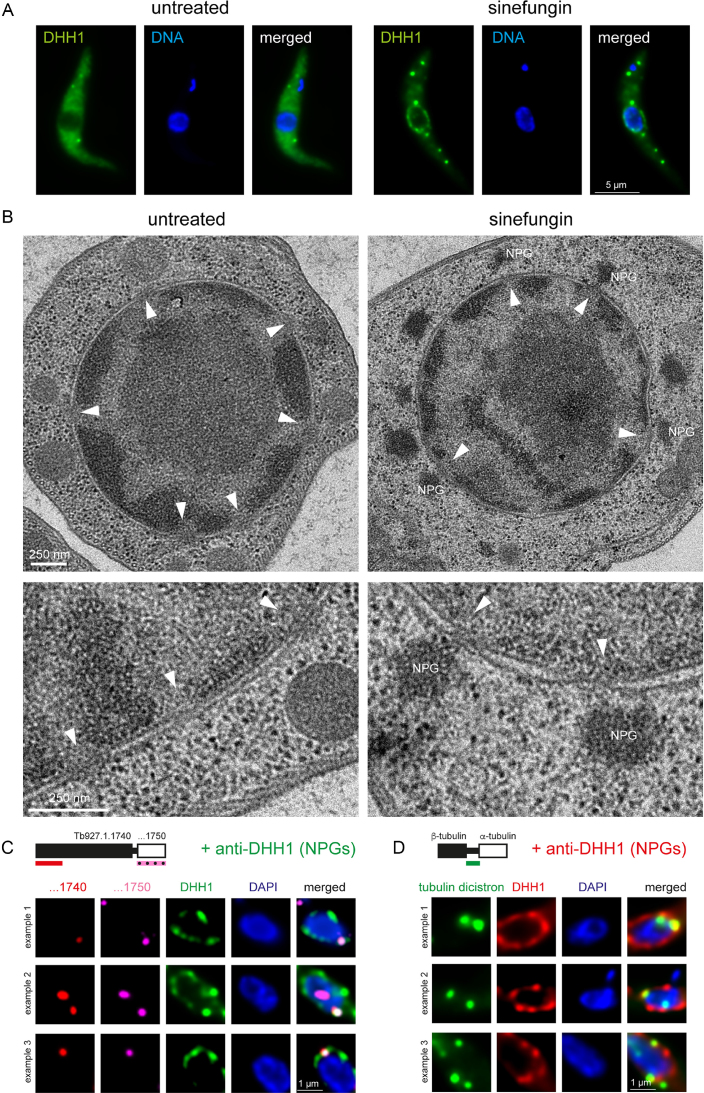
NPGs are at the nuclear pores and colocalize with unspliced mRNAs. (**A**) Cells expressing eYFP-DHH1 as a marker protein for NPGs from the endogenous locus (23) were imaged untreated or after sinefungin treatment. One representative cell is shown. DNA is stained with DAPI. (**B**) Electron microscopy images of Epon-embedded cells treated with and without sinefungin. Nuclear pores are marked with white triangles and NPGs (electron-dense structures close to the nuclear periphery) are labelled. The cells were induced to overexpress SCD6-eYFP for 24 h (26) prior to imaging. Further images of sinefungin treated cells are shown in Supplementary Figure S8. (**C** and **D**) smFISH of the indicated transcripts (compare Figure 4) was combined with immunofluorescence staining of the NPG marker protein DHH1. For each transcript, three examples of unspliced transcripts co-localizing with NPGs are shown (single plane images of a deconvolved Z-stack).

Inhibition of trans-splicing by either sinefungin or transfection of a morpholino oligo antisense to the U2 snRNA causes both preferential localization of unspliced transcripts to the nuclear periphery (Figure 4B and Supplementary Figure S7) and the localization of several RNA-binding proteins, including DHH1, to NPGs (23) (Figure 5A). To investigate possible connections between NPGs and the unspliced mRNA molecules, we first analysed the localization of NPGs in more detail using electron microscopy. In wild-type cells, no electron dense structures were visible at the nuclear periphery after sinefungin treatment. Therefore, we used a cell line which 3-fold overexpressed the NPG protein SCD6 (a highly conserved Lsm domain protein), resulting in an increase in the fraction of proteins that localize to NPGs (26). With this cell line, electron dense areas were clearly observed at the nuclear pores in cells treated with sinefungin, but not in untreated cells (Figure 5B and Supplementary Figure S8). To confirm that these electron dense patches are indeed NPGs, we did immuno-gold labelling for the NPG marker protein SCD6, in both wild-type cells and SCD6-overexpressing cells. Sinefungin treated cells revealed clusters of gold-grains at the nuclear periphery that were absent in untreated cells (Supplementary Figure S9). This was true for both wild-type cells and SCD6-overexpressing cells, but the latter also showed electron dense structures at the clusters of the gold-grains. Thus, the electron microscopy studies strongly suggest that NPGs are localized at the outside of nuclear pores.

Next, immunofluorescence staining of the NPG marker protein DHH1 was combined with smFISH against two of the unspliced mRNA investigated above (Figure 4). We observed co-localization of unspliced transcripts with NPGs (Figure 5C and D). It was not possible to examine whether all unspliced transcripts at the nuclear periphery co-localized with NPGs, or only a subset as not all NPGs could be visualized due to the protease digestion and severe chemical treatment required for the FISH procedure.

In conclusion, inhibition of trans-splicing causes the formation of NPGs at the outside of nuclear pores and these granules contain unspliced transcripts.

### Nuclear periphery granules resemble trypanosome stress granules in protein composition

**Figure 6.**
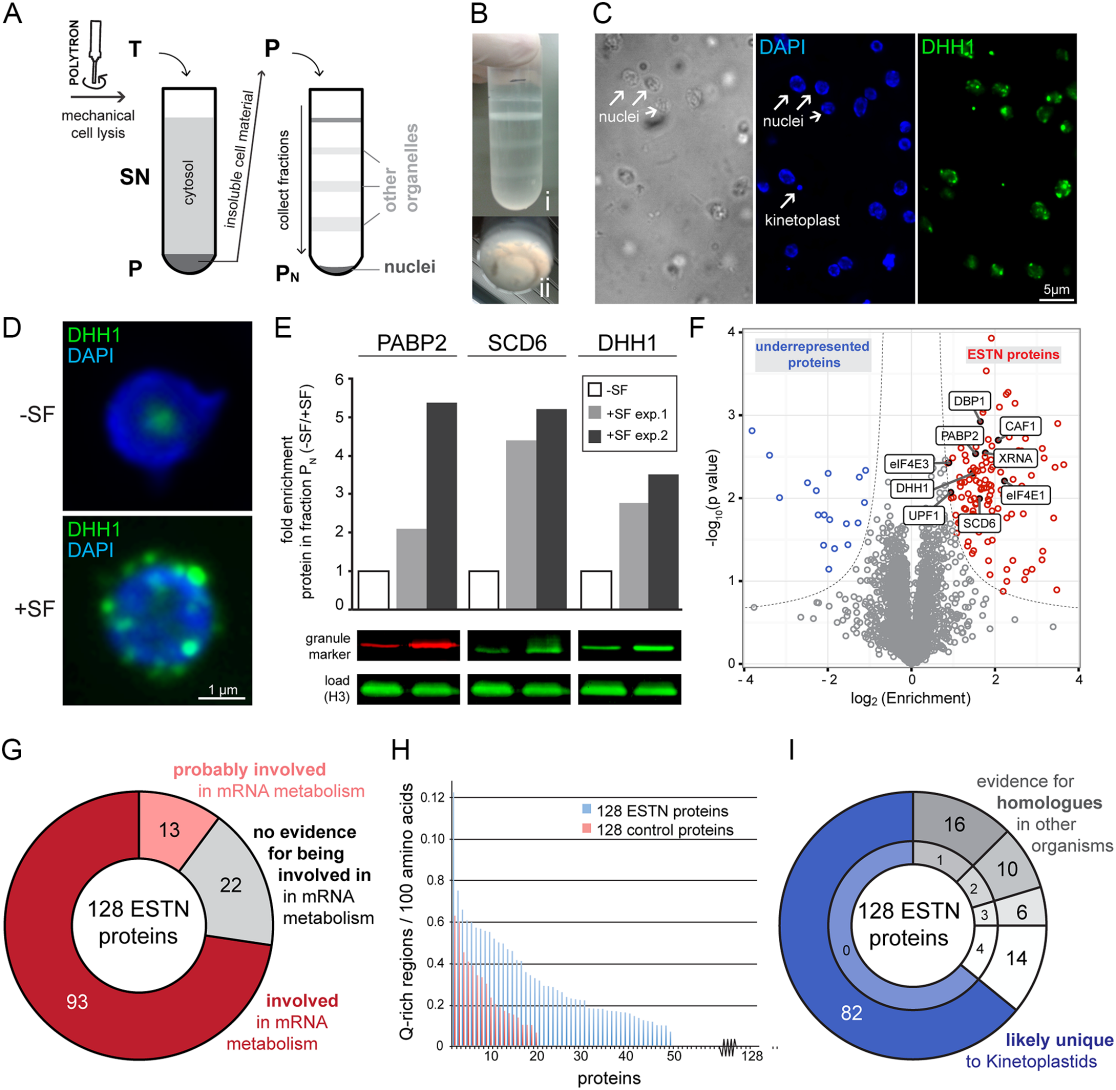
Purification of nuclei with attached NPGs. (**A**) Schematics of the procedure. Cells expressing the NPG marker protein eYFP-DHH1 were incubated in the presence and absence of sinefungin (SF) and mechanically lysed with a POLYTRON^®^ mixer. The total cell lysate (T) was separated on a sucrose cushion into a pellet (P) containing crude cell material (nuclei, flagella and kinetoplasts) and a supernatant (SN) containing mostly soluble material from the cytosol. The pellet was resuspended, loaded to a three-step sucrose gradient and further separated by ultracentrifugation into soluble fractions and the pellet fraction P_N_. (**B**) Photograph of the three-step sucrose gradient after ultracentrifugation. The different layers of the gradient (i) and the small ring-shaped precipitate at the bottom (Pellet P_N_) (ii) are shown. (**C**) Microscopic analysis of Pellet P_N_ of sinefungin treated cells. Isolated nuclei are clearly visible as ovoids and only few other structures are present, such as remnants of flagella (brightfield image). Nuclei are intact (native shape, nucleolus is visible by absence of DAPI staining) and P_N_ contains few kinetoplasts (DAPI image). The presence of NPGs is clearly visible (DHH1). Fluorescence images are shown as deconvolved Z- projections, the phase image is a single plane image. (**D**) The presence of NPGs is specific to purified nuclei from sinefungin treated cells. Z-projections of representative nuclei isolated from untreated cells (-SF) and sinefungin treated cells (+SF) are shown. (**E**) NPG marker proteins are enriched in fraction P_N_ of sinefungin-treated cells. The NPG marker proteins PABP2, SCD6 and DHH1 were quantified from fraction P_N_ of control cells (-SF) and NPG-induced cells (+SF) by western blot. For each protein, the fold-enrichment upon sinefungin treatment from two independent experiments is plotted, and one representative western blot is shown. Histone H3 (H3) served as a loading control. (**F**) Volcano plot of the mass spectrometry data. ESTN proteins are shown in red, proteins that were underrepresented in nuclei of sinefungin treated cells are shown in blue and proteins with no enrichment are shown in grey. The nine previously known NPG proteins are labelled. (**G**) Most ESTN proteins are involved in mRNA metabolism. Proteins were classified as involved in mRNA metabolism, when they either had a known function in mRNA metabolism (shown experimentally or by being a homologue to a protein with a known function in mRNA metabolism), or possessed RNA-binding domains. Proteins were also classified as involved, when two independent lines of literature evidence support a function in mRNA metabolism. Proteins were classified as ‘probably involved’ in mRNA metabolism, if evidence was based on only one such literature evidence. More details are in Supplementary Table S1A. (**H**) Enrichment of Q-rich regions in the ESTN proteins. The number of Q-rich regions per 100 amino acids is shown for the 128 ESTN proteins and for a randomly chosen group of control proteins. Q-rich regions were quantified from NPG proteins and a randomly chosen group of control proteins based on the method described in (70,71). Briefly, amino acid sequences of all proteins were divided in 60-mers and a 60-mer was defined as Q-rich region, if it contained at least seven glutamate residues. For each protein, the number of Q-rich regions normalized to the length of the protein (Q-rich regions / 100 amino acids) is blotted; for a better visualization, the proteins were sorted according to their enrichment in Q-rich regions. The control proteins were proteins encoded by adjacent genes on chromosome 1 ranging from Tb427.01.100 to Tb427.01.3280. (**I**) Most ESTN proteins are unique to Kinetoplastids. All ESTN proteins were screened for putative homologues in *Saccharomyces cerevisiae* (taxid4932), *Caenorhabditis elegans* (taxid6239), *Mus musculus* (taxid10090) and *Plasmodium falciparum* (taxid5833) by BLAST (default parameters). The top hit of the BLAST analysis was then used for a reverse BLAST against the *T. brucei* genome. If the original protein was the top hit in the reciprocal BLAST approach and had an e-value of <1 × 10^−5^, the protein was classified as having a homologue in the respective organism. With these definitions, the majority of proteins were unique to *T. brucei* (blue). The remaining proteins had putative homologues in either one, two, three or four of the four organisms (grey). The complete dataset is shown in Supplementary Table S1D.

To further characterize NPGs, we determined the granule’s proteome by isolating nuclei of untreated and sinefungin treated cells with a mechanical method for cell lysis that prevents NPG dissociation (31,32). The procedure is schematically shown in Figure 6A and B: after mechanical cell lysis, the insoluble material (mainly nuclei and cytoskeleton) is pelleted through a sucrose cushion, resuspended and applied to a discontinuous sucrose gradient. The pelleted fraction contained the purified nuclei with only little contaminations (32) and NPGs remained attached to the nuclei during the purification; this was monitored using the fluorescent NPG marker protein eYFP-DHH1 expressed from the endogenous locus (Figure 6C and D). Furthermore, the NPG marker proteins PABP2, SCD6 and DHH1 were between 2- and >5-fold enriched in the isolated nuclei of sinefungin treated cells when compared to untreated cells, indicating a successful enrichment of NPGs (Figure 6E). Protein samples of the purified nuclei from untreated and sinefungin treated cells from three biological replicates were analysed by mass spectrometry. About 3195 protein groups were detected, corresponding to ∼^1^/_3_ of all proteins encoded by the *T. brucei* genome (5–7) (Supplementary Table S1C). In total 128 proteins were significantly (FDR <0.05) enriched in purified nuclei of sinefungin treated cells in comparison to nuclei of untreated cells and thereafter called ESTN proteins (enriched in sinefungin-treated nuclei) (Figure 6F; Supplementary Table S1A). These included all nine previously known NPG proteins (23) (labelled in Figure 6F), indicating a successful co-purification of NPGs with the nuclei. 18 proteins were underrepresented in nuclei purified from sinefungin treated cells (Supplementary Table S1B); these were not further analysed in this study.

**Figure 7.**
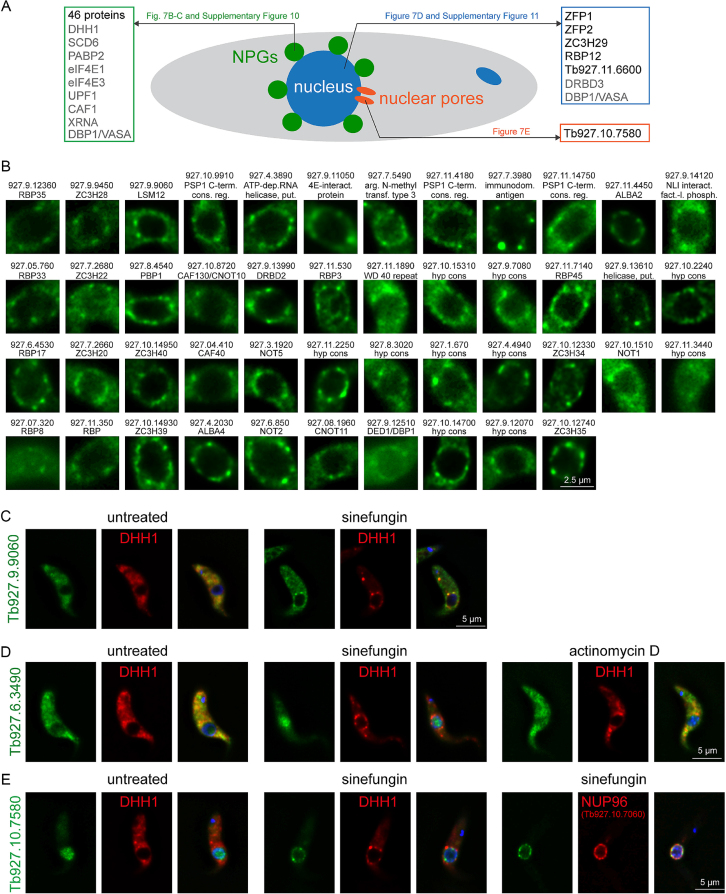
Validation of ESTN proteins by localization of eYFP fusion proteins. ESTN proteins were expressed as eYFP fusion proteins in a cell line expressing the NPG marker protein mChFP-DHH1; all expressions were from the endogenous locus. (**A**) Summary of all ESTN proteins that change localization in response to sinefungin, either to the nucleus, to NPGs, or to nuclear pores. Proteins that were localized previously (23) are written in grey colour, proteins localized in this work are written in black colour with reference to the respective figure(s). (**B**) ESTN proteins that re-localize to NPGs in response to sinefungin treatment. For each protein, one representative nucleus with NPGs is shown. Images of complete cells, including co-localization with mChFP-DHH1 and DAPI staining, are shown in Supplementary Figure S10. Images are either single plane images of a deconvolved Z-stack or Z-projections (details in Supplementary Table S1A). Names and gene identifiers are shown, excluding ‘Tb.’. (**C**) Example image of an ESTN protein that relocalizes to NPGs with sinefungin. (**D**) Example image of an ESTN protein that relocalizes to the nucleus with sinefungin, but not with actinomycin D. (**E**) The ESTN protein Tb927.10.7580 relocalizes to the nuclear pores upon sinefungin treatment: it does not co-localize with the NPG marker protein DHH1, but with the nuclear pore protein *Tb*NUP96.

As expected for RNA granule proteins, the 128 ESTN proteins were mostly involved (73%) or probably involved (10%) in mRNA metabolism (Figure 6G), and Q-rich regions were overrepresented (Figure 6H). Notably, an unusually large fraction of 64% was unique to Kinetoplastids (Figure 6I). Ten of the 128 ESTN proteins had previously been localized in the presence of sinefungin (23) (grey text-colour in Figure 7A). The localization of the remaining 118 ESTN proteins in the absence and presence of sinefungin was investigated by fusion to eYFP and expression from the endogenous locus in a cell line also expressing mChFP-DHH1 as a marker for NPGs. For 52 proteins, sinefungin treatment caused an unequivocal change in localization. The majority of these (46 proteins) co-localized with mChFP-DHH1 to NPGs and were mainly cytoplasmic in the absence of sinefungin (Figure 7B and C and Supplementary Figure S10). Six proteins changed their localization from the cytoplasm to the nucleus, either completely (ZFP1, ZFP2 and RBP12) or partially (ZC3H29 and Tb927.11.6600); these changes did not occur in the presence of actinomycin D, indicating they are caused by the inhibition of splicing and not by the absence of newly synthesized mature mRNAs (Figure 7D and Supplementary Figure S11). One protein (Tb927.10.7580) re-localized from the nucleoplasm to the nuclear pores, as evidenced by co-localization with NUP96-mChFP (Figure 7E). For eight proteins, sinefungin caused no change in localization (Supplementary Figure S12). We were not able to analyse the remaining 58 proteins as eYFP tagging was either not successful (15 proteins) or gave too low a signal to unequivocally define or exclude NPG formation (43 proteins). In total, we have identified a list of 113 candidate NPG proteins and validated the localization for 55 proteins by eYFP tagging (Supplementary Table S1).

NPGs differ significantly from trypanosome starvation stress granules in localization and sensitivity to transcriptional and translational inhibitors (23). However, a comparison of the NPG proteome with the recently published proteome of stress granules (30), that also considered experimental data from many studies (details in Supplementary Table S1A) and from a recent genome wide localization study TrypTag (53) revealed remarkable similarities between the two granule types (Supplementary Figure S13). In particular, there are no data from localization experiments suggesting a certain protein may be specific to either granule type (Supplementary Figure S13). Moreover, the majority of translation initiation factors, including the proteins that are currently considered the major components of the eIF4F complex, eIF4E4 and eIF4G3, (54) are absent from both granule types (Supplementary Figure S13).

NPGs were previously suggested to be related to perinuclear germ granules of adult gonads that are best studied in *Caenorhabditis elegans* (55), based on similarities in localization, sensitivity to actinomycin D and insensitivity to cycloheximide (23). However, we found no similarities in protein contents between the two granule types: the majority of known germ granule proteins have no homologues in *T. brucei*; vice versa, the majority of validated NPG proteins have no homologues in *C. elegans* (Supplementary Table S1D). Moreover, inhibition of trans-splicing in *C. elegans* with spliceostatin A (56) did not cause any altered localization of the P-granule marker proteins CGH-1 or PGL-1 to perinuclear granules in either gonads or somatic cells of young adult worms (data not shown).

In conclusion, we have described and validated a subset of the proteome of trypanosome NPGs, which shows no apparent differences to the proteome of starvation stress granules. NPGs contain most of the cytoplasmic RNA-binding proteins, consistent with the granules being the result of unspliced transcripts reaching out of the nuclear pores, bound to the standard set of RNA-binding proteins.

## DISCUSSION

In the current universal model, an mRNA needs to be fully processed to be transported to the cytoplasm, which includes the completion of transcription, splicing, capping and adenylation. Several quality control systems act in parallel to ensure that only fully mature mRNAs can exit the nucleus; and these control systems can only be bypassed under extreme stress conditions (57,58). Here, we have analysed mRNA export in the early-branched eukaryote *T. brucei*. Consistent with the absence of homologues to most proteins involved in nuclear export control in opisthokonts, we found that trypanosomes can start to export mRNAs, even though transcription and polyadenylation have not yet been completed. To the best of our knowledge, this is the first report of co-transcriptional nuclear export in any eukaryote. Still, a nuclear export control appears to be present that slows or prevents the completion of nuclear export of unspliced, polycistronic transcripts, causing their accumulation into granules at the cytoplasmic side of the nucleus.

We have adapted our recently established intramolecular multi-colour smFISH (39) to analyse nuclear export in trypanosomes. We have previously studied transcription and decay (39) and now extended the method to visualize mRNAs crossing the nuclear border. The use of a very large endogenous reporter mRNA allows to directly visualize processes of mRNA metabolism that are too transient to be observed with an average-sized mRNA. Still, it needs to be kept in mind that most mRNAs are much smaller and in particular co-transcriptional export may be a rare event in average-sized mRNAs with their much shorter transcription time. Thus, the data show that a checkpoint to efficiently prevent co-transcriptional export is lacking in trypanosomes, not so much that the majority of transcripts actually exits this way. Importantly, we confirmed key findings of this work by experiments that do not rely on the large reporter RNA. In particular, we show the enrichment of dicistronic RNAs at the nuclear periphery and colocalization with NPGs also for average-sized mRNAs (Figures 4B and 5D). A second essential tool used in this study was the global inhibition of trans-splicing. Currently, there is no good alternative that allows the detection of unspliced transcripts of average-abundant mRNAs, but it needs to be kept in mind that such a global interference with mRNA metabolism could potentially result in a shortage in certain splicing or export factors. As a consequence, the pathway taken by the unspliced transcripts that accumulate after sinefungin treatment may differ from the pathway followed by the small amount of unspliced transcripts that accumulate naturally. Still, at least for the abundant tubulin RNA we were able to show leakage of a small amount of tubulin dicistron to the cytoplasm and accumulation to the nucleus and nuclear periphery in Figure 4A, even in the presence of splicing. These data confirm that at least some partially spliced transcripts can reach the cytoplasm and thus circumvent any nuclear export control pathway. Moreover, the key finding of the paper, the fact that nuclear export can be co-transcriptional, is also independent on inhibition of trans-splicing.

How is unspliced, polycistronic mRNA prevented from nuclear export? mRNAs can be actively retained in the nucleus by marking them as immature (retention model), or, mRNAs are actively selected as export-competent by proteins that recognize certain features of matureness (selection model). In higher eukaryotes, there is evidence for both systems acting in parallel, to ensure an even tighter control system (3). Our data argue against the presence of an active selection system in trypanosomes, as the start of export is clearly not prevented if the mRNA is immature. Even the finding that the RNA exosome preferentially removes unspliced transcripts (15) does not necessarily mean an active selection for decay of such transcripts, but could be the result of these transcripts being retained in the nucleus and thus being longer accessible to the RNA exosome (59). Instead, there are two lines of evidence for the retention model: (i) small unspliced tubulin mRNAs (dicistrons) are preferentially exported to the cytoplasm in comparison to larger polycistrons ((23) and Supplementary Figure S6 this work): dicistrons only have one intergenic region and thus have fewer binding sites for retention factors in comparison to larger polycistrons; and (ii) unspliced transcripts start nuclear export but appear stuck inside the nuclear pores, the easiest explanation for this is a retention mechanism, possibly by factors that recognize intronic sequences at the nuclear basket.

**Figure 8.**
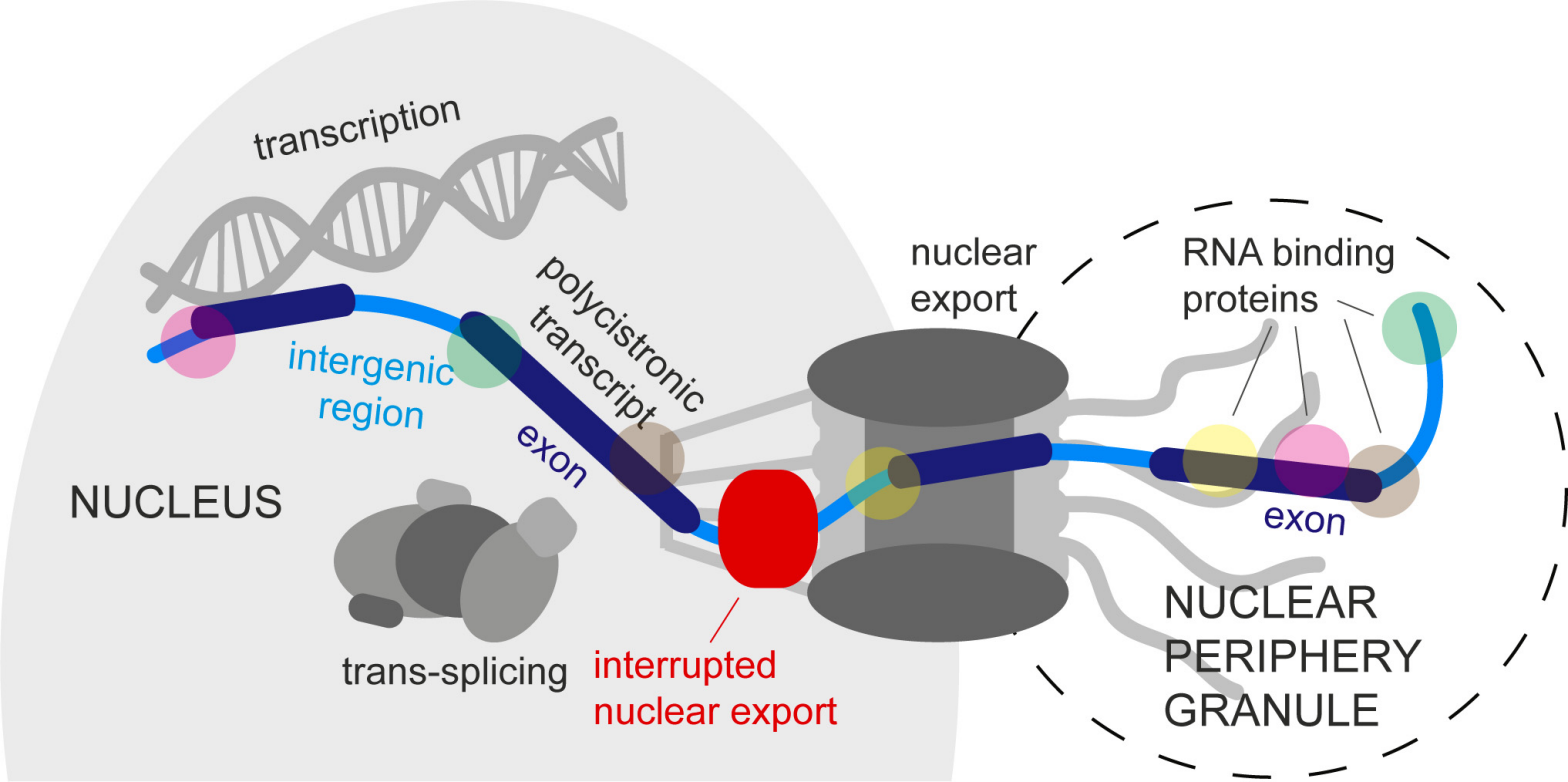
Proposed model of mRNA export in trypanosomes. The figure shows a polycistronic transcript that is unspliced and therefore retained in the nuclear pore. The 5′ end of the polycistron has been exported and localizes to NPGs. See text for more details.

The following scenario of nuclear mRNA metabolism in trypanosomes is in best agreement with all available data (Figure 8). mRNAs are transcribed as long polycistrons; the transcription rate is unknown, but models predict that between 0.05 to 0.5 mRNA molecules are synthesized per minute in each cell (60). Processing occurs by co-transcriptional trans-splicing the capped spliced leader RNA and polyadenylation of the upstream gene; the majority of mRNAs will be processed within a few minutes of synthesis (61). Many mRNAs are likely fully mature, before they reach the nuclear pores and require no further control system. However, individual splice sites differ significantly in efficiency (60), possibly mediated by differences in nucleosome density across exon borders that influence the speed of RNA polymerase II (62). Thus, some unspliced transcripts are present in the nucleoplasm. A fraction of such unspliced transcripts is 3′-5′ degraded by the nuclear RNA exosome (15), another fraction can be processed into mature mRNA by conventional splicing at an unknown location (24). Both, decay and processing, are likely in competition with nuclear export: Unlike in opisthokonts, unspliced trypanosome mRNAs can start nuclear export; whether this is dependent on the presence of a 5′ cap remains to be investigated. Some unspliced transcripts reach the cytoplasm, but in many cases the transcripts are prevented from the completion of nuclear export and accumulate in the nuclear pores. It is possible that such a retention of nuclear export may allow enough time for the spliceosome to complete mRNA processing or for the RNA exosome to complete decay; perhaps the retention is caused by spliceosomal or exosomal factors being recruited to intergenic regions of the mRNA at the nuclear basket. We have identified several proteins as a ‘side-product’ of NPG purification that change their localization from the cytoplasm to the nucleus or from the nucleoplasm to the nuclear pores upon inhibition of trans-splicing (Figure 7D and E, Supplementary Figure S11); these are promising candidates for proteins acting in nuclear export control at the nuclear basket. If splicing is globally inhibited, the number of unprocessed mRNAs that are stuck in the nuclear pores becomes so large, that the exported 5′ ends with their associated RNA-binding proteins become visible as NPGs.

mRNA export control was so far mainly studied in opisthokonts, which represent only a small fraction of eukaryotic diversity; and even in these organisms it remains poorly understood. Trypanosomes are not the only protozoa that have no or very divergent mRNA export control factors (1). Apicomplexa, for example, which transcribe and process their mRNAs conventionally from promoters and by cis-splicing, have a very diverge functional homologue to Mex67 with almost no sequence similarities to its opisthokont counterpart (63); many further nuclear export control proteins are diverse or absent too (64,65). The solutions that have evolved to avoid the production of aberrant proteins from immature mRNAs thus appear diverse and adapted to the specific needs of the organism, in quality and mechanism. Trypanosomes have virtually no introns and this may be the reason why they can afford a certain leakage of unspliced mRNAs to the cytoplasm. The use of recently advanced *in vitro* (39), *in vivo* (66) and *in silico* (67) techniques to study nuclear export should result in a more comprehensive and comparative picture of nuclear export control mechanisms across the range of eukaryotes.

## DATA AVAILABILITY

The mass spectrometry proteomics data have been deposited to the ProteomeXchange Consortium via the PRIDE partner repository with the dataset identifier PXD010143 (68,69).

## Supplementary Material

Supplementary Data
