# Supplementary material for: Trypanosomes can initiate nuclear export co-transcriptionally
Source: Nucleic Acids Res. 2018 Nov 12;47(1):266–82. doi: 10.1093/nar/gky1136 (PMC6326799; doi:10.1093/nar/gky1136)
Supplement: Supplementary Data [file gky1136_supplemental_files.zip › SupFigures_1.pdf]

Supplementary Figure 1

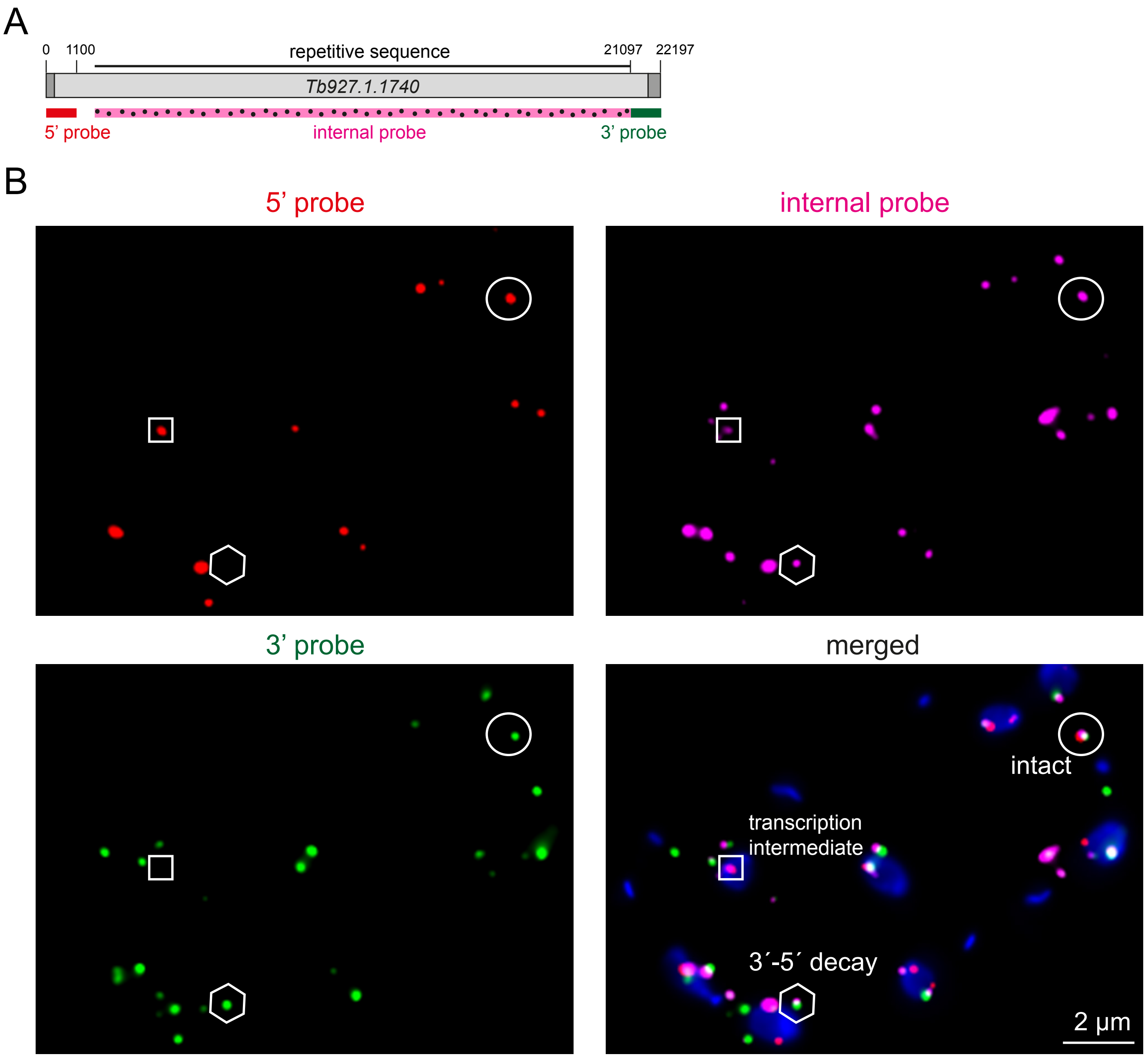

Supplementary Figure 1: Intra-molecular 3 colour smFISH of Tb927.1.7140
Cells were stained for Tb927.1.7140 by intra-molecular 3 colour smFISH as indicated in **(A)**. One image is shown **(B)** and examples of intact mRNA molecules (red-pink-green), 5´-3´ decay intermediates (pink-green) and transcription intermediates (red-pink) are marked. Note that the staining efficiency varies between the different molecules; the reason is that the amplification of the signal by the FISH method (Affymetrix, branched DNA technology) is very space-consuming and labelling efficiency thus differs, dependent on how protein-rich the environment of an mRNA molecule is. For quantification, contrast and brightness of the images in this paper were therefore adapted each individual spot to best visibility; this is not visible in this figure. DNA is stained with DAPI (blue).

Supplementary Figure 2

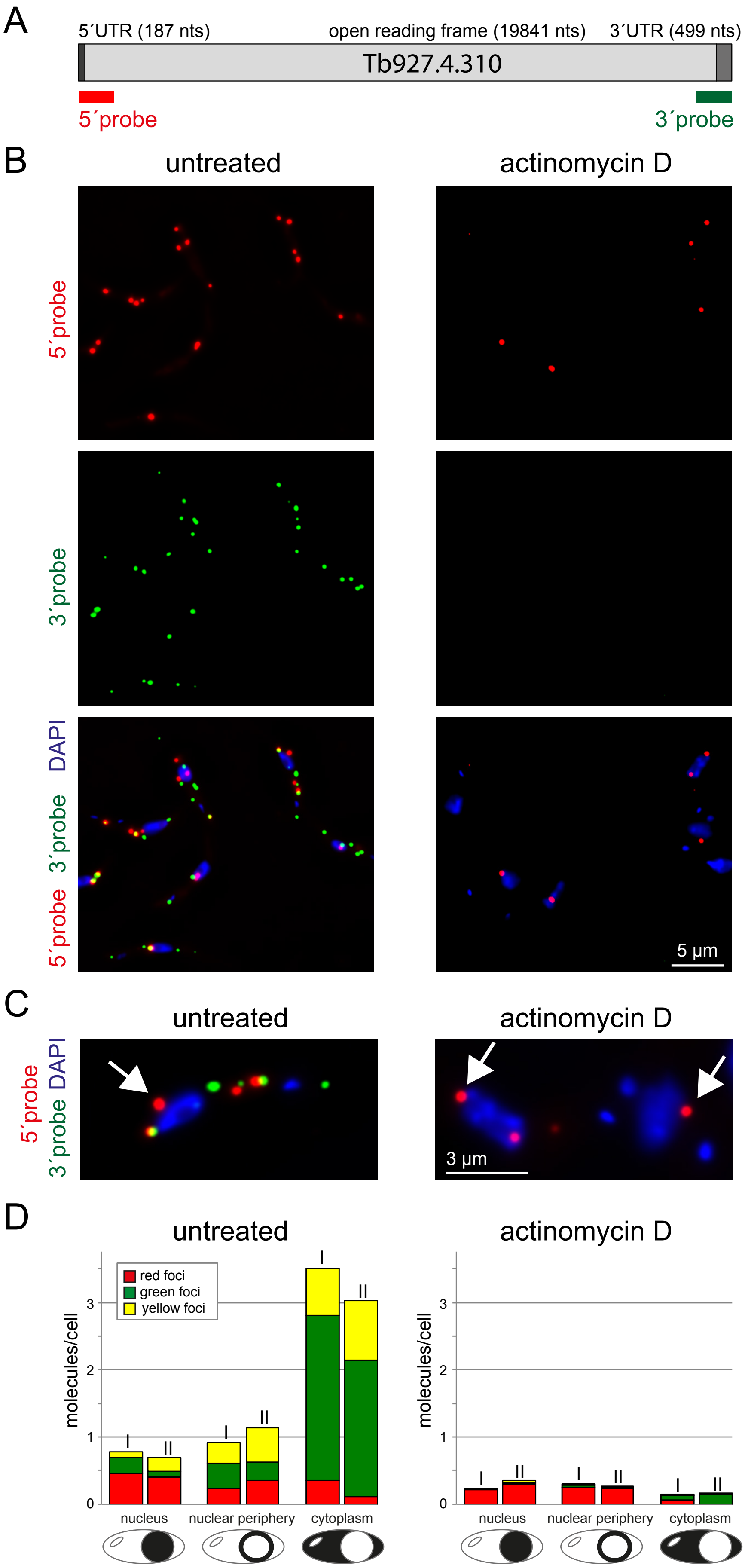

Supplementary Figure 2: Two colour intramolecular smFISH of Tb927.4.310.
Wild type cells were left untreated or treated with actinomycin D and probed for Tb927.4.310 by intramolecular 2-colour smFISH as indicated in **(A)**. Images of many cells are shown **(B)**. Selected inlets of these images are shown enlarged **(C)**, with arrows pointing to mRNAs that are putatively in co-transcriptional nuclear export (enriched in actinomycin D treated cells). Quantifications of two experiments (I and **II**, each with 100 cells) are shown in (**D**).

Figure S3

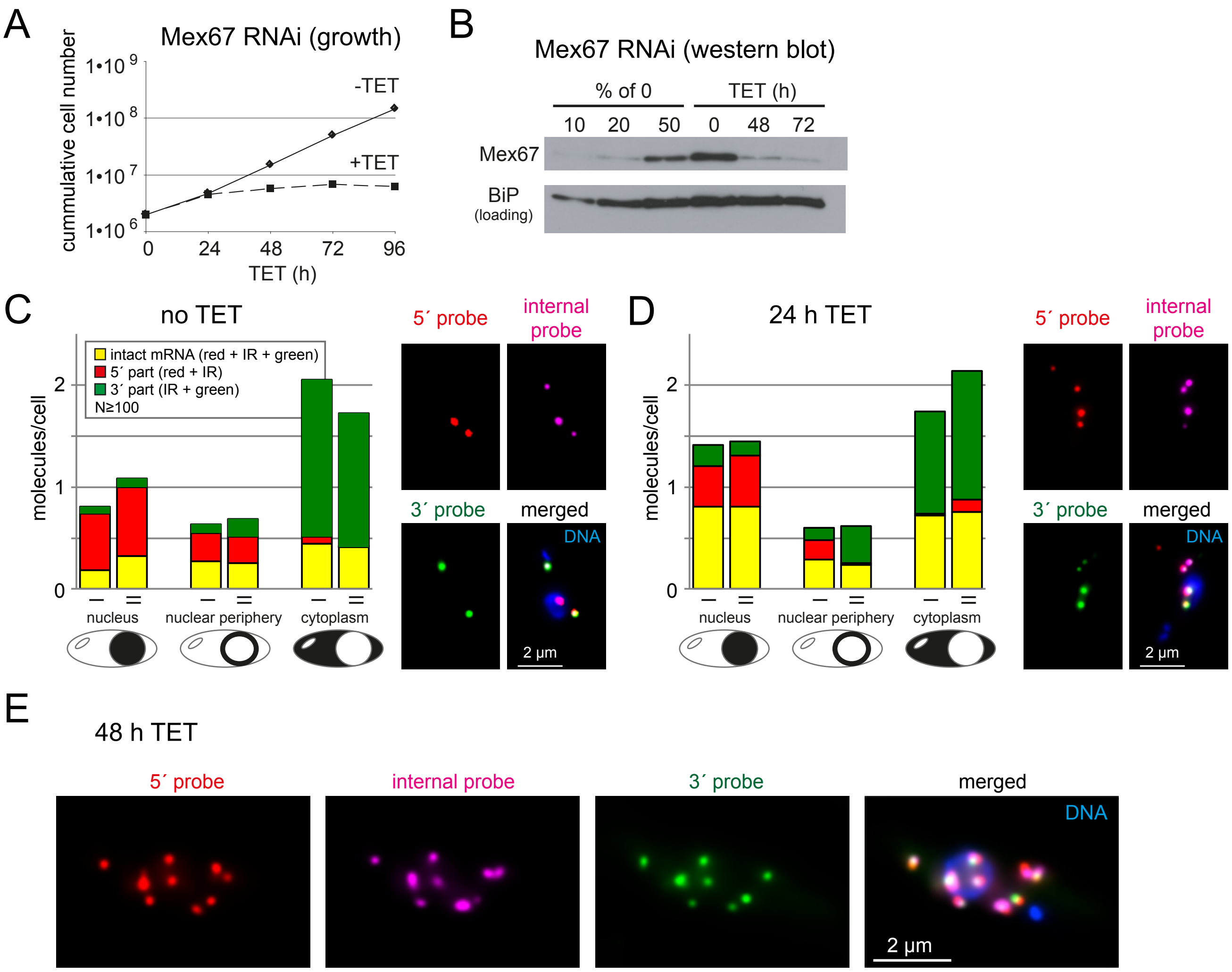

Supplementary Figure 3: Three colour intramolecular smFISH of Tb927.1.1740 following RNAi depletion of MEX67

RNAi depletion of MEX67 was induced by tetracycline (TET). **(A)** Growth curve: growth arrest starts at 24 hours of RNAi induction. Representative data of one clone of two are shown. **(B)** Western blot with samples taken during RNAi depletion of MEX67 were probed with specific antiserum to TbMEX67 and BiP as a loading control. 10, 20 and 50% of the sample from non-induced cells was loaded to estimate the decrease in protein. Mex67 protein was reduced to about 20% within 48 hours of RNAi depletion. Representative data of one clone of two are shown. **(C-D)** smFISH: Non-induced cells and cells depleted for MEX67 for 24 hours (24h TET) were analysed as in Figure 3A. Data of two experiments (**I** and **II**) are shown. With Mex67 depleted, we did observe the expected increase in intact mRNA molecules in the nucleus and, importantly, we did not observe any change in the number of mRNA molecules at the nuclear periphery. We also observed an increase in intact mRNA molecules in the cytoplasm, suggesting inhibition of mRNA decay as a result of impaired nuclear export; this was not further investigated in this study. **(E)** Mex67 RNAi depletion is lethal and causes severe morphological changes that prevent a meaningful analysis of the smFISH data after 48 hours induction. However, there is a clear further increase in intact mRNAs in the nucleus and we did not observe any obvious accumulation of mRNAs at the nuclear periphery at this time-point. One example cell is shown.

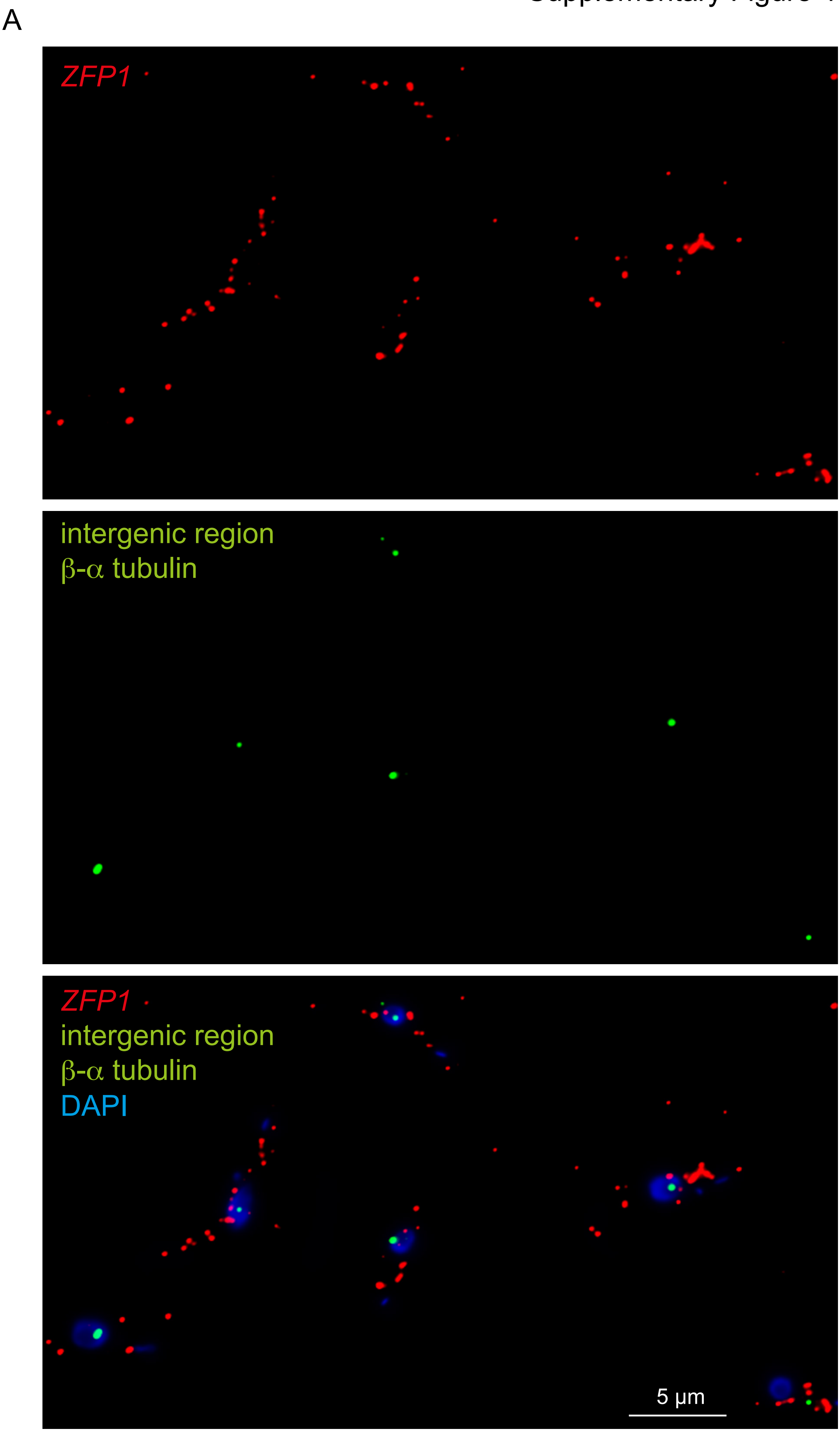
A
ZFP1
intergenic region
β-α tubulin
ZFP1
intergenic region
β-α tubulin
DAPI
5 μm

B

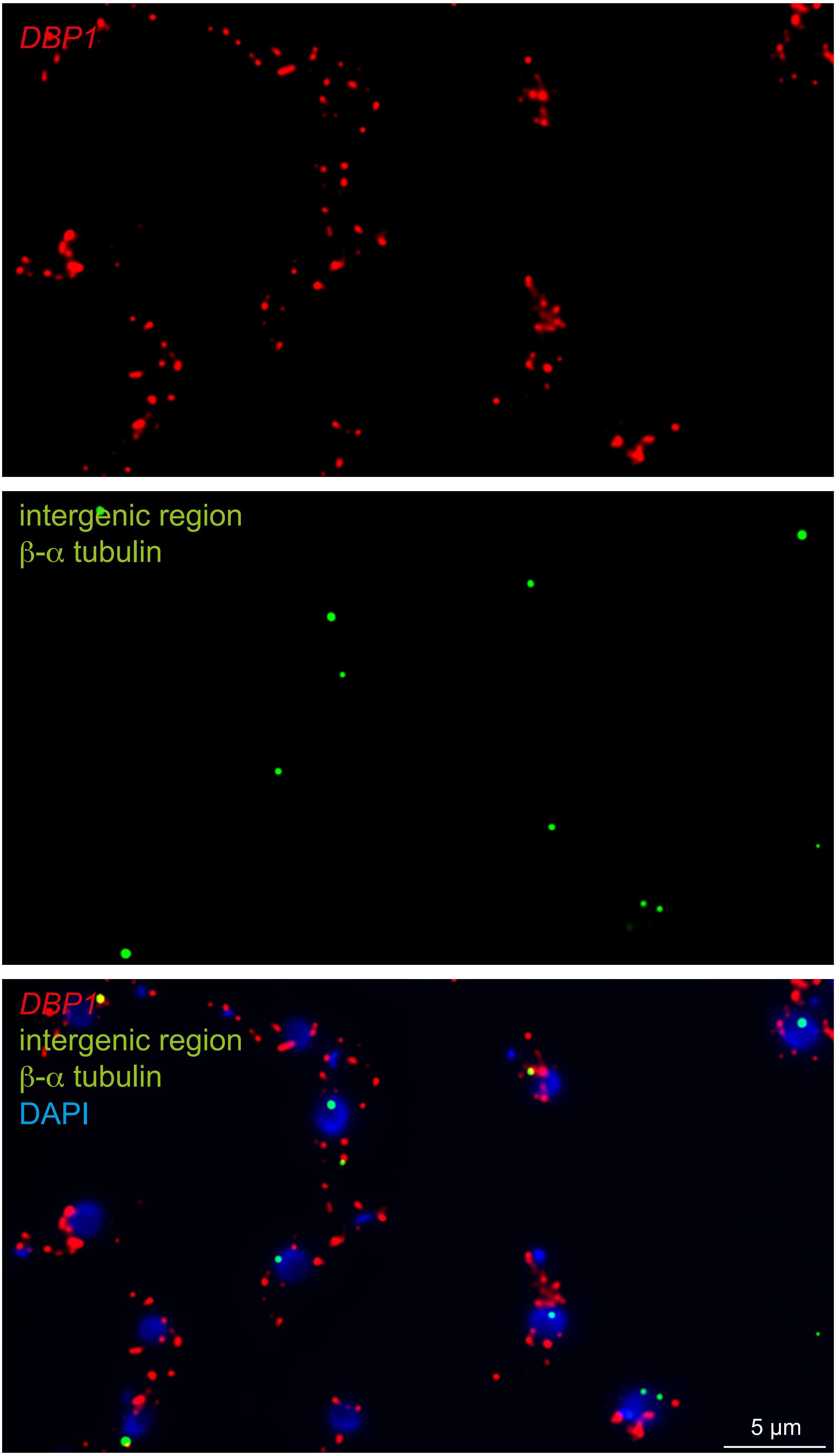
DBP1
intergenic region
β-α tubulin
DBP1
intergenic region
β-α tubulin
DAPI
5 µm

Supplementary Figure 4: Mature mRNAs are not enriched in the nucleus
Cells were probed with smFISH probes antisense to the intergenic region between β and α tubulin in green and antisense to the open reading frame sequences of *ZFP1* (A) or *DBP1* (B) in red. While the unspliced tubulin RNA is enriched in the nucleus and at the nuclear periphery, both the *ZFP1* and the *DBP1* mRNAs are preferentially cytoplasmic.

Supplementary Figure 5

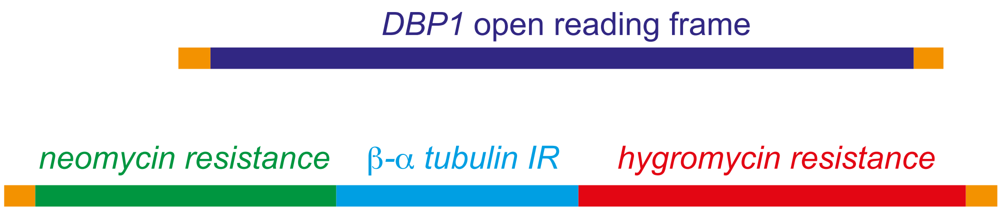

Supplementary Figure 5: Replacement of the *DBP1* locus by the neomycin and hygromycin resistance genes.
The wild type *DBP1* gene is shown on top, and the modified locus, used to transcribe the neo-hyg dicistron is shown underneath. The regions shown in orange are the 80 nucleotides flanking the *DBP1* open reading frame that were used for homologous recombination: One *DBP1* allele was replaced with the NEO-HYG cassette, while the second allele remained wild type.

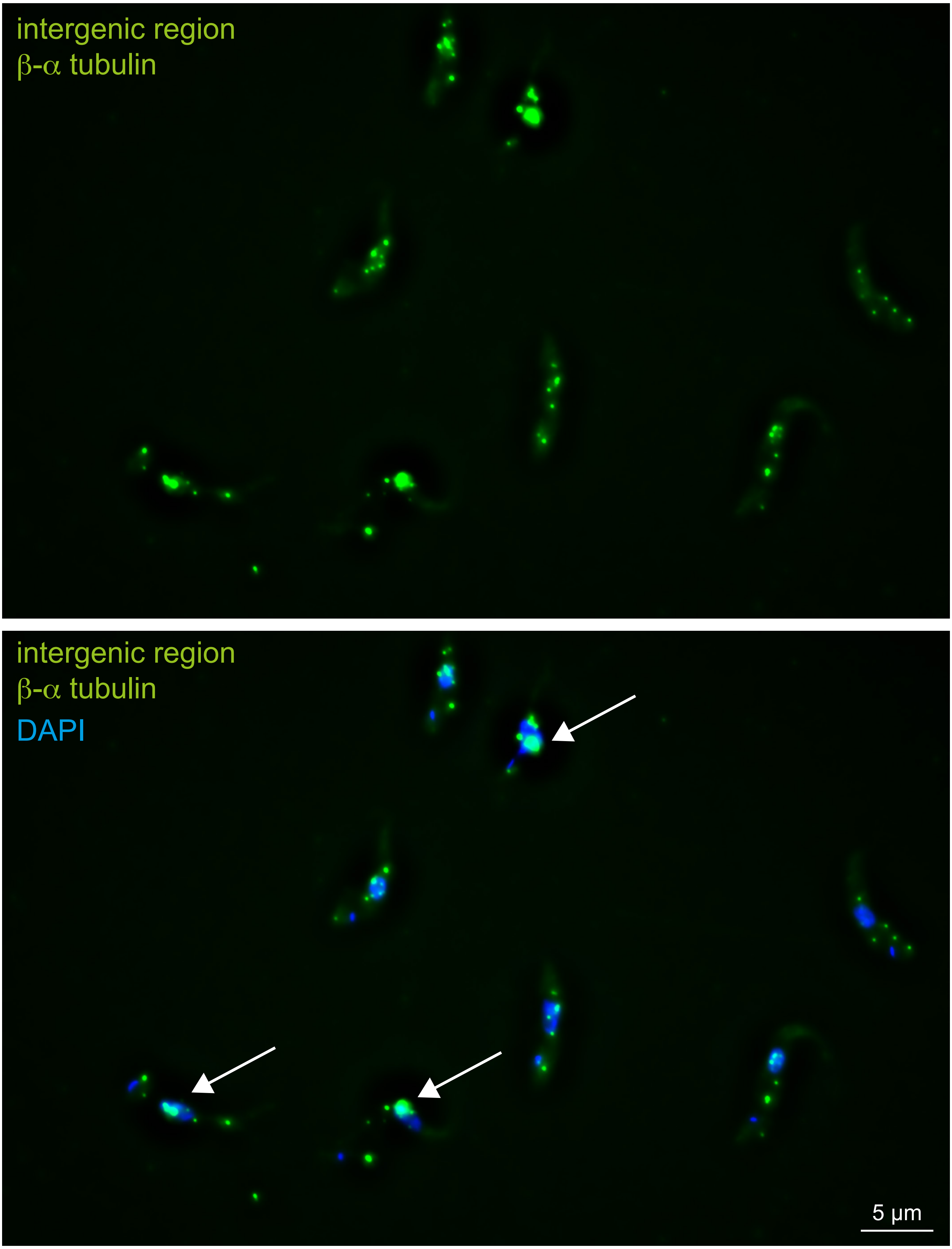

Supplementary Figure 6: Detection of polycistronic tubulin mRNAs by smFISH after inhibition of trans-splicing
Cells were treated with sinefungin for 60 minutes to inhibit trans-splicing and probed by smFISH for the intergenic region between α and β tubulin (green). The DNA is stained with DAPI. Inhibition of trans-splicing causes the formation of multicistronic tubulin mRNAs and the larger molecules are often enriched in the nucleus (arrows).

Supplementary Figure 7

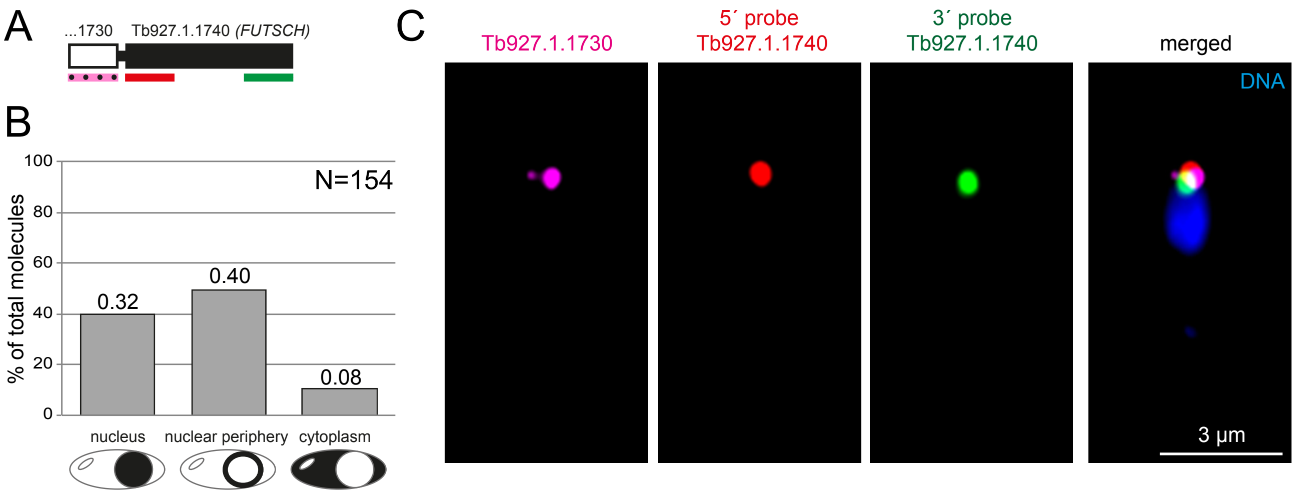

Supplementary Figure 7: Inhibition of trans-splicing by a morpholino antisense to the trypanosome U2 snRNA

Trans-splicing was inhibited by transfecting wild type trypanosome cells with a morpholino antisense to the U2 snRNA.

(A) mRNAs of two adjacent genes were probed by three-colour smFISH as indicated on the schematics.

(B) The localisation of unspliced mRNAs (foci stained with all three colours) was determined. The bars show the percentage of mRNAs localised to the nucleus, the nuclear periphery or to the cytoplasm. The numbers on top of the bars are the molecules per cell (average values). These numbers are a little lower in comparison to sinefungin treated cells, probably because the efficiency of inhibition of splicing is lower when using the morpholino. Transfection with the control morpholino did not result in inhibition of trans-splicing: there were only 6 foci with three colours in 169 cells, 5 of which were in the nucleus and thus likely transcription intermediates prior to trans-splicing.

(C) Example image of a cell that was transfected with the U2 snRNA morpholino and then stained for unspliced mRNAs with the three probes indicated in A. In this cell, the unspliced mRNA is localised at the nuclear periphery.

Supplementary Figure 8

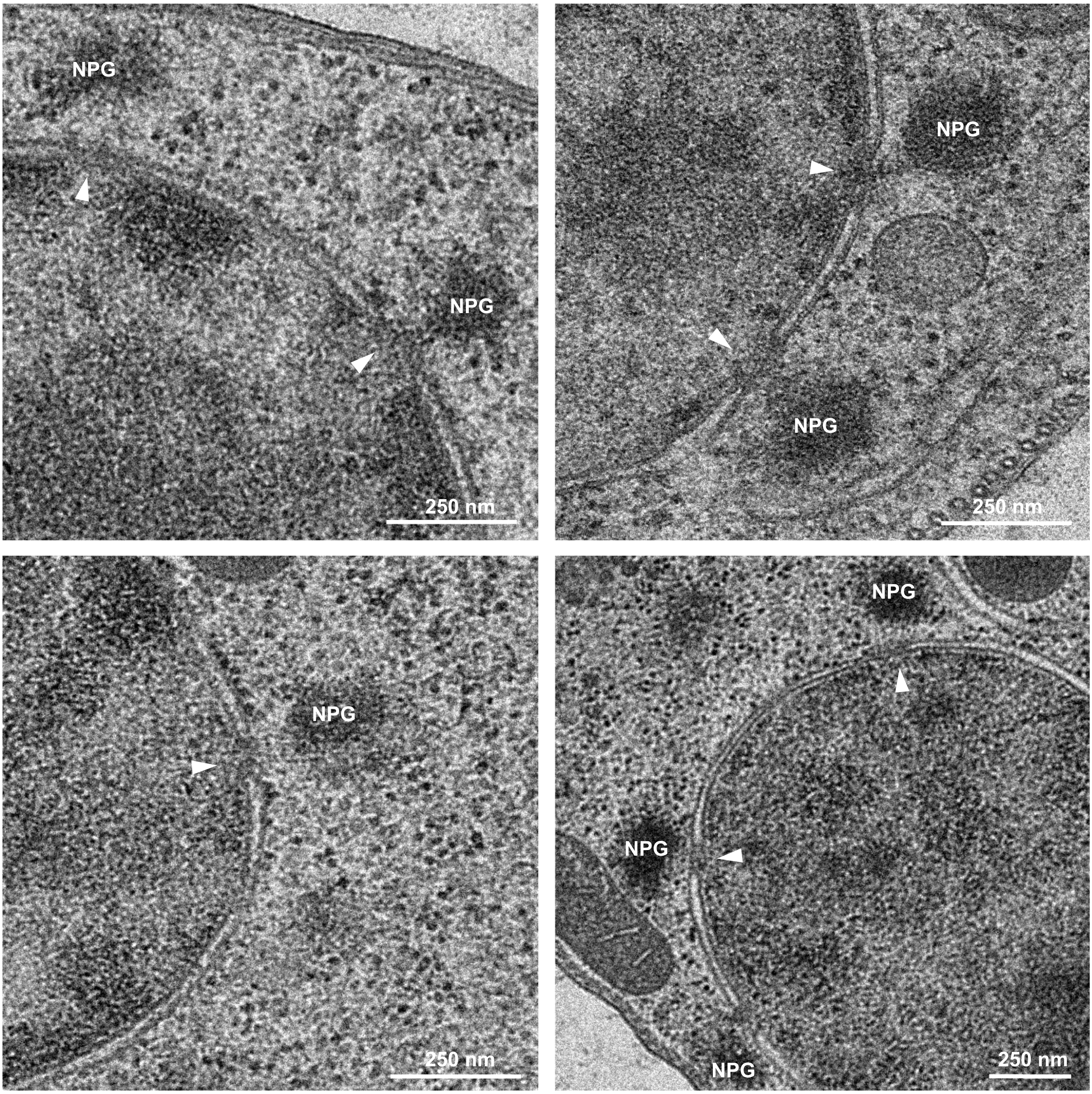

Supplementary Figure 8: NPGs are localised outside of nuclear pores
Cells were induced to overexpress SCD6 to obtain better granule structures and treated with sinefungin for NPG induction. Electron microscopy images of Epon-embedded cuts are shown. NPGs are labelled and nuclear pores are marked with triangles.

Supplementary Figure 9

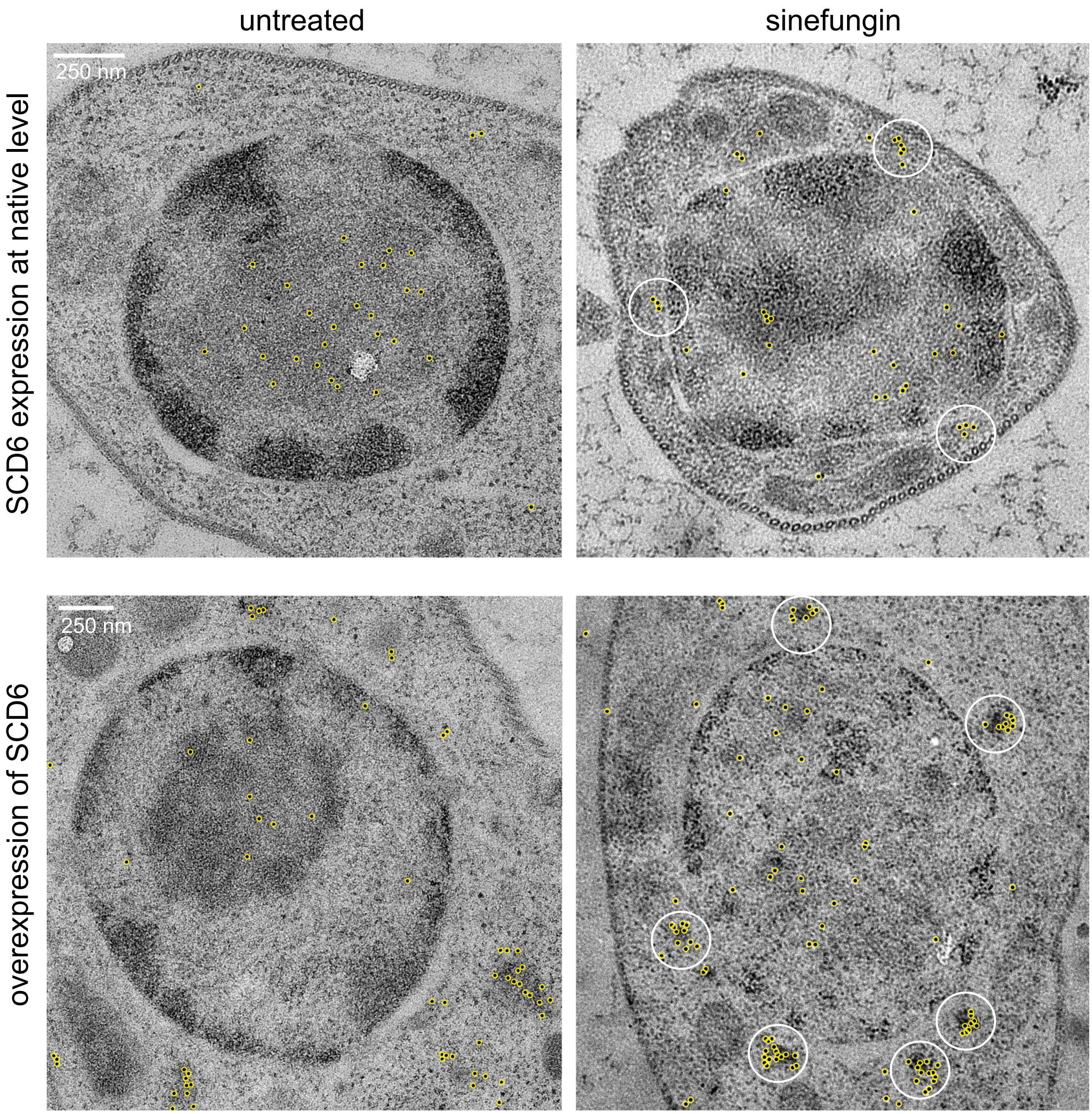

Supplementary Figure 9: Detection of NPGs by immunogold-labelling
Immunogold labelling of wild type cells (upper panel) and cells overexpressing SCD6 (lower panel) with anti-SCD6. Gold grains are labelled with yellow circles and accumulations of gold grains at the nuclear periphery (NPGs) are encircled white. Note that the nuclear pores are not visible with the LR white embedding method.

Supplementary Figure 10

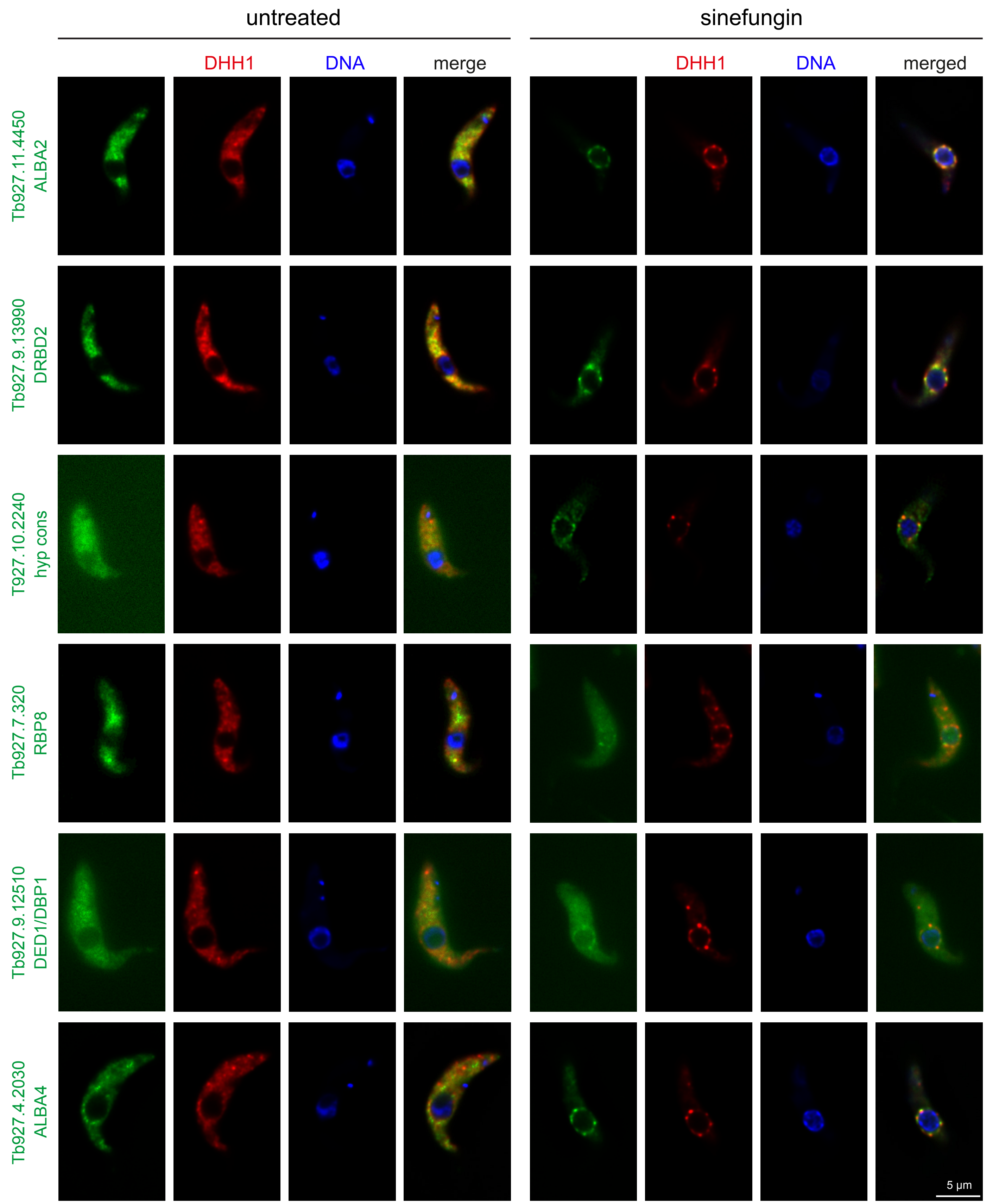

Supplementary Figure 10

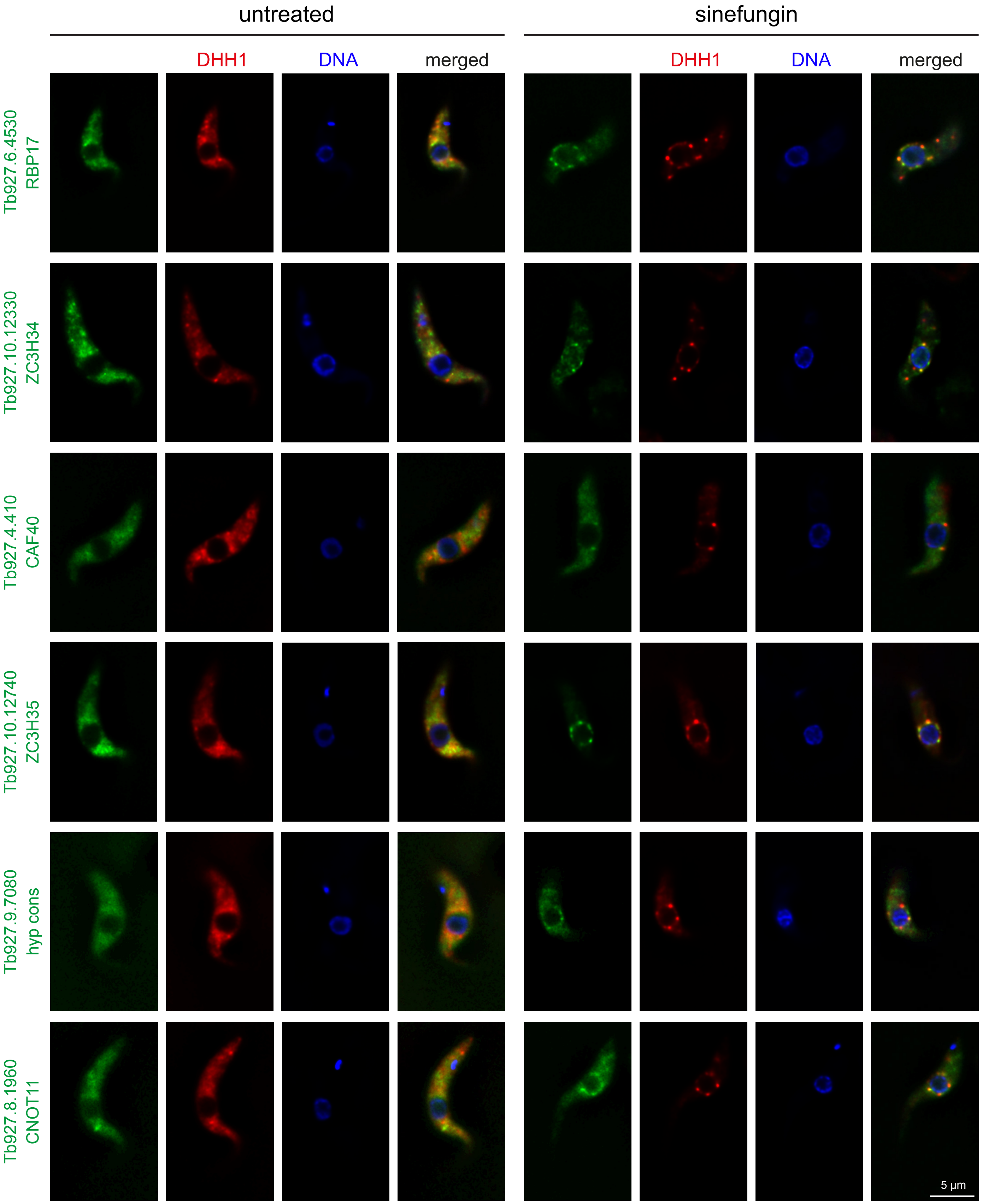

Supplementary Figure 10

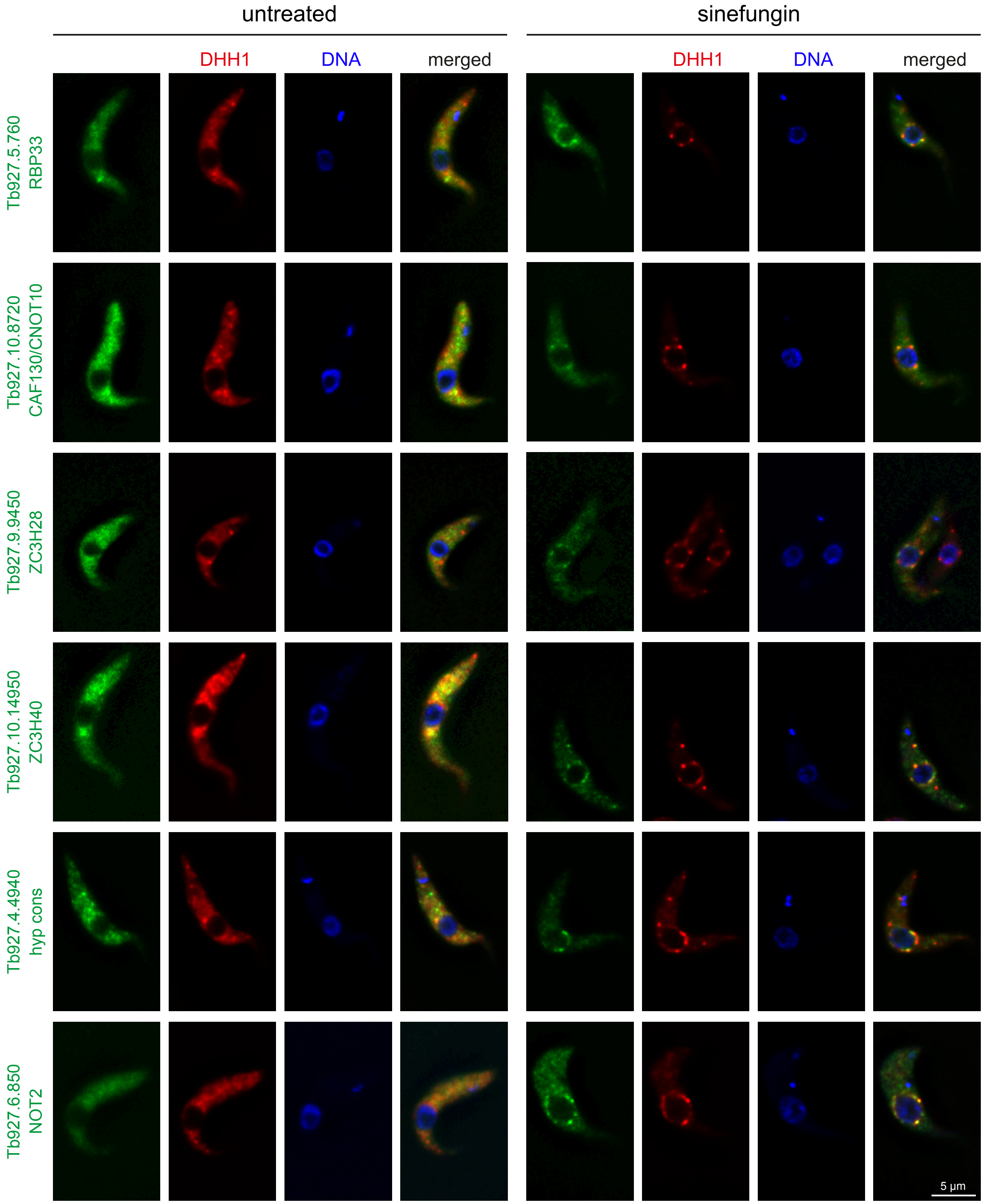

Supplementary Figure 10

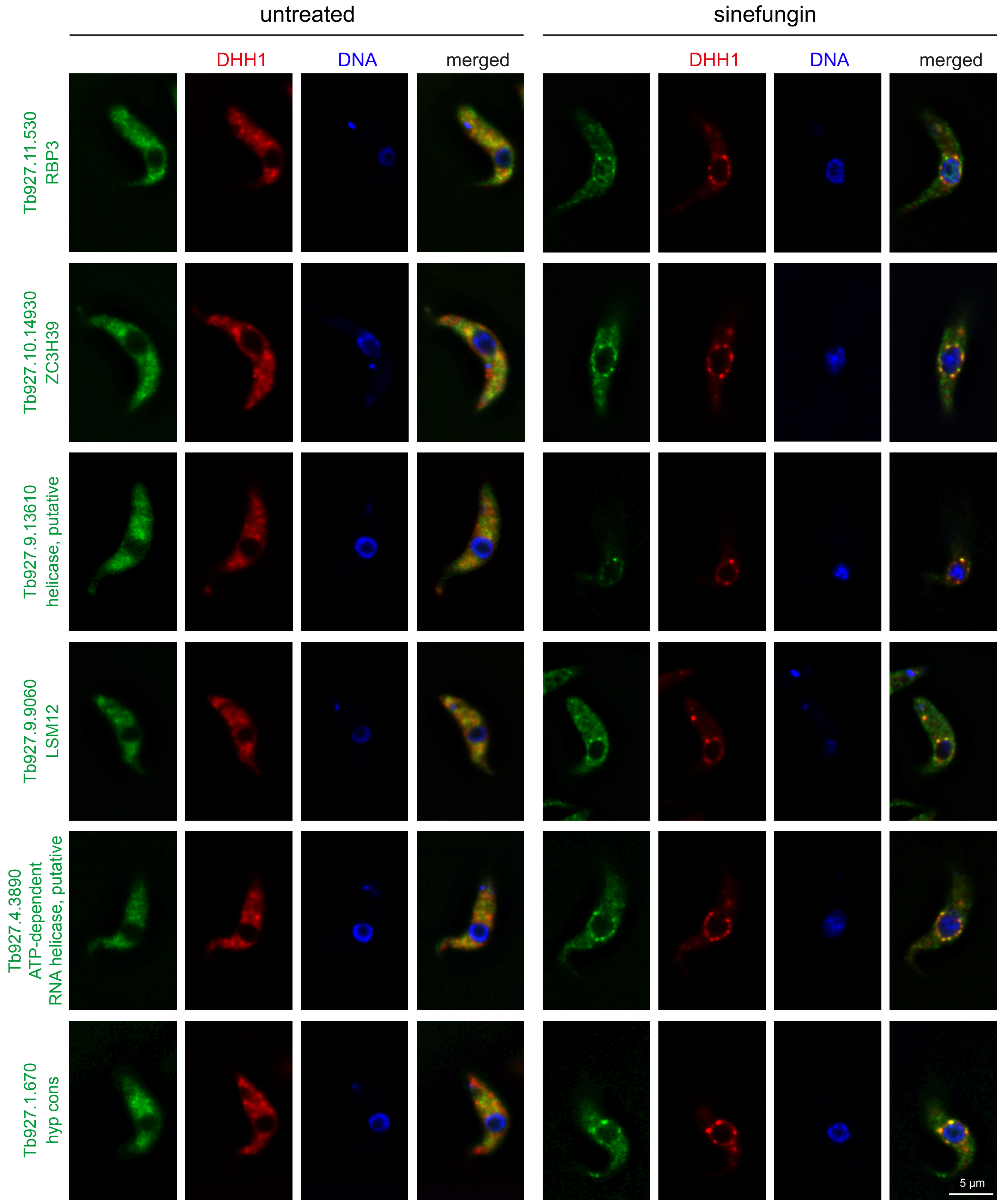

Supplementary Figure 10

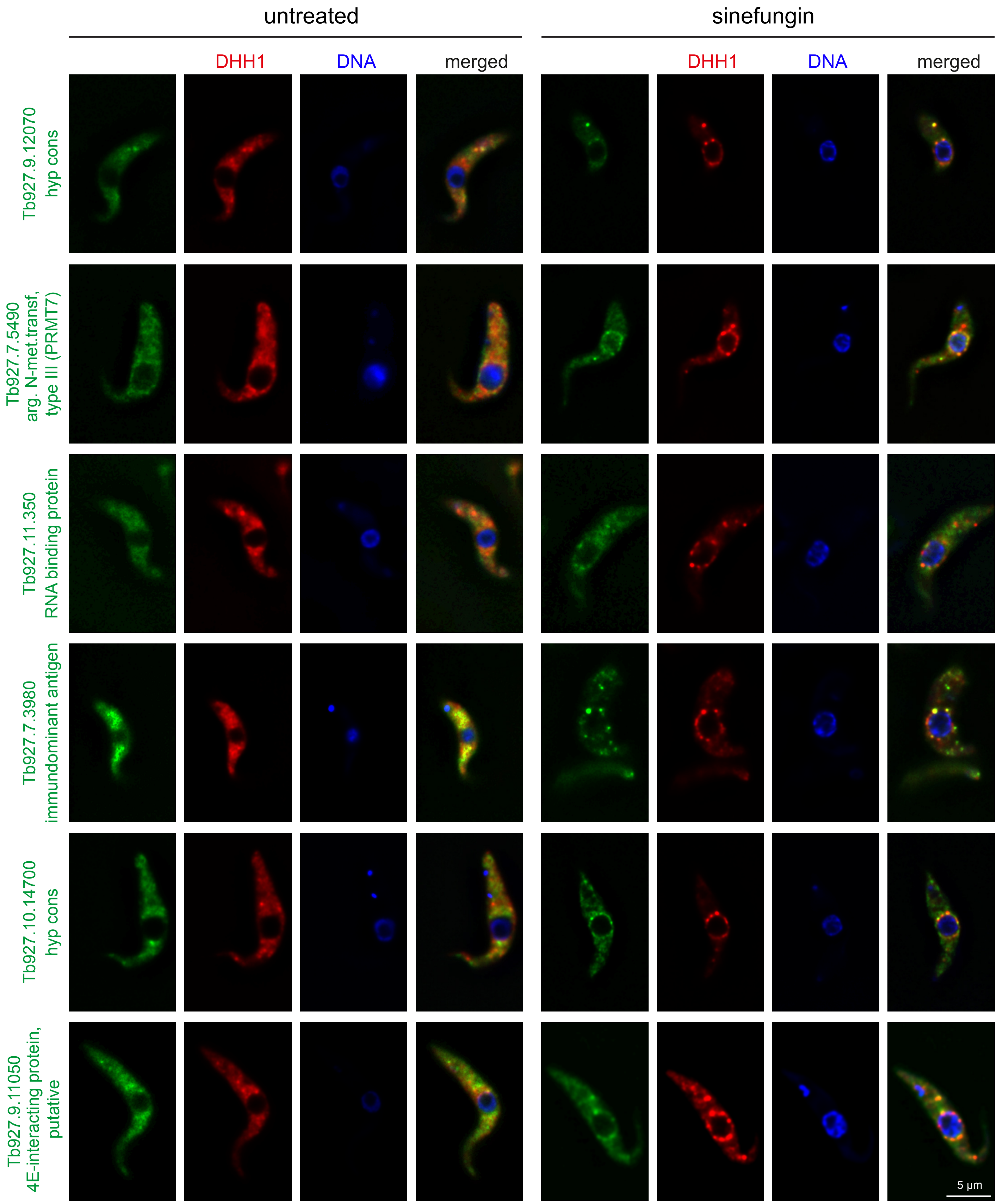

Supplementary Figure 10

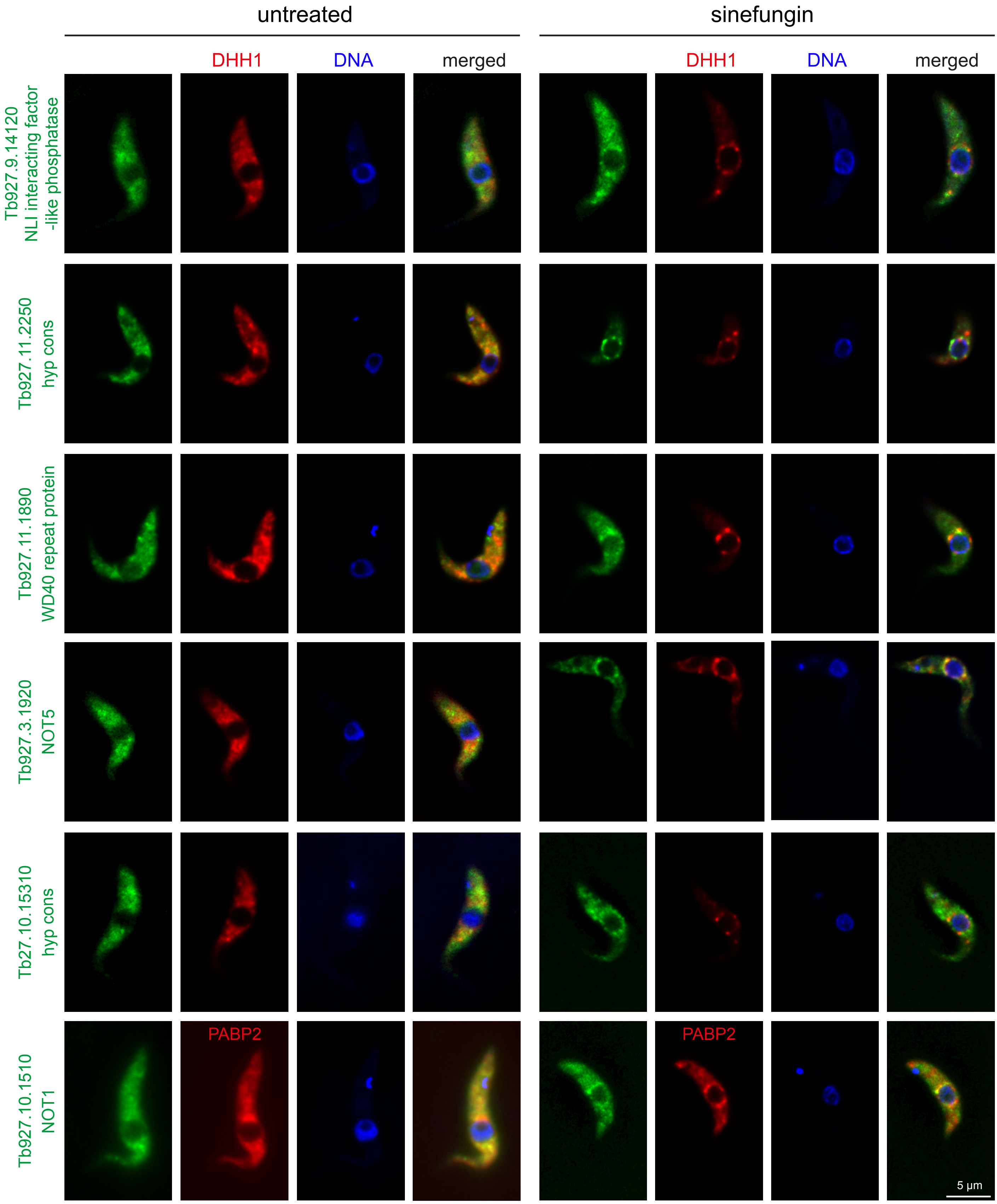

Supplementary Figure 10

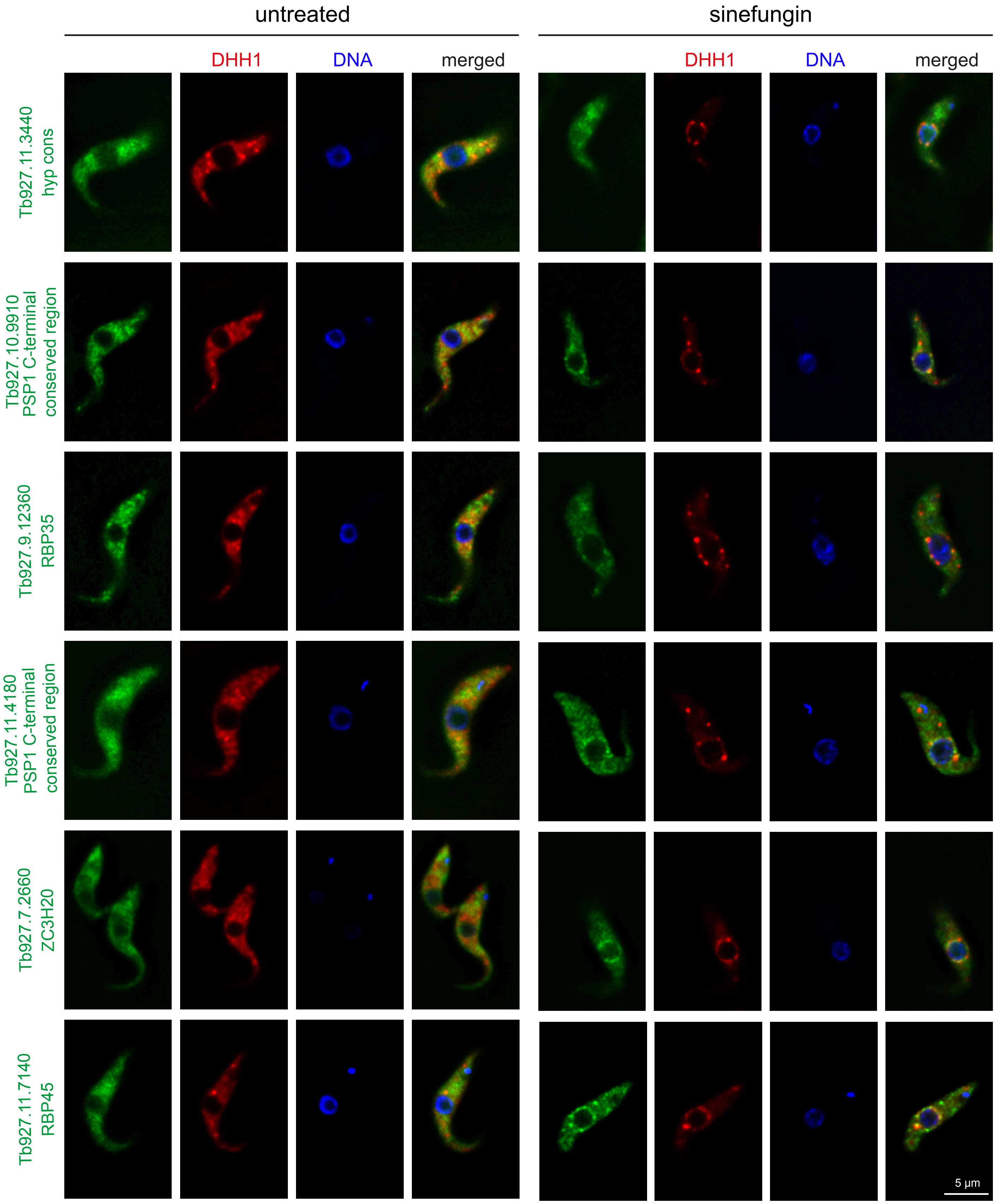

Supplementary Figure 10

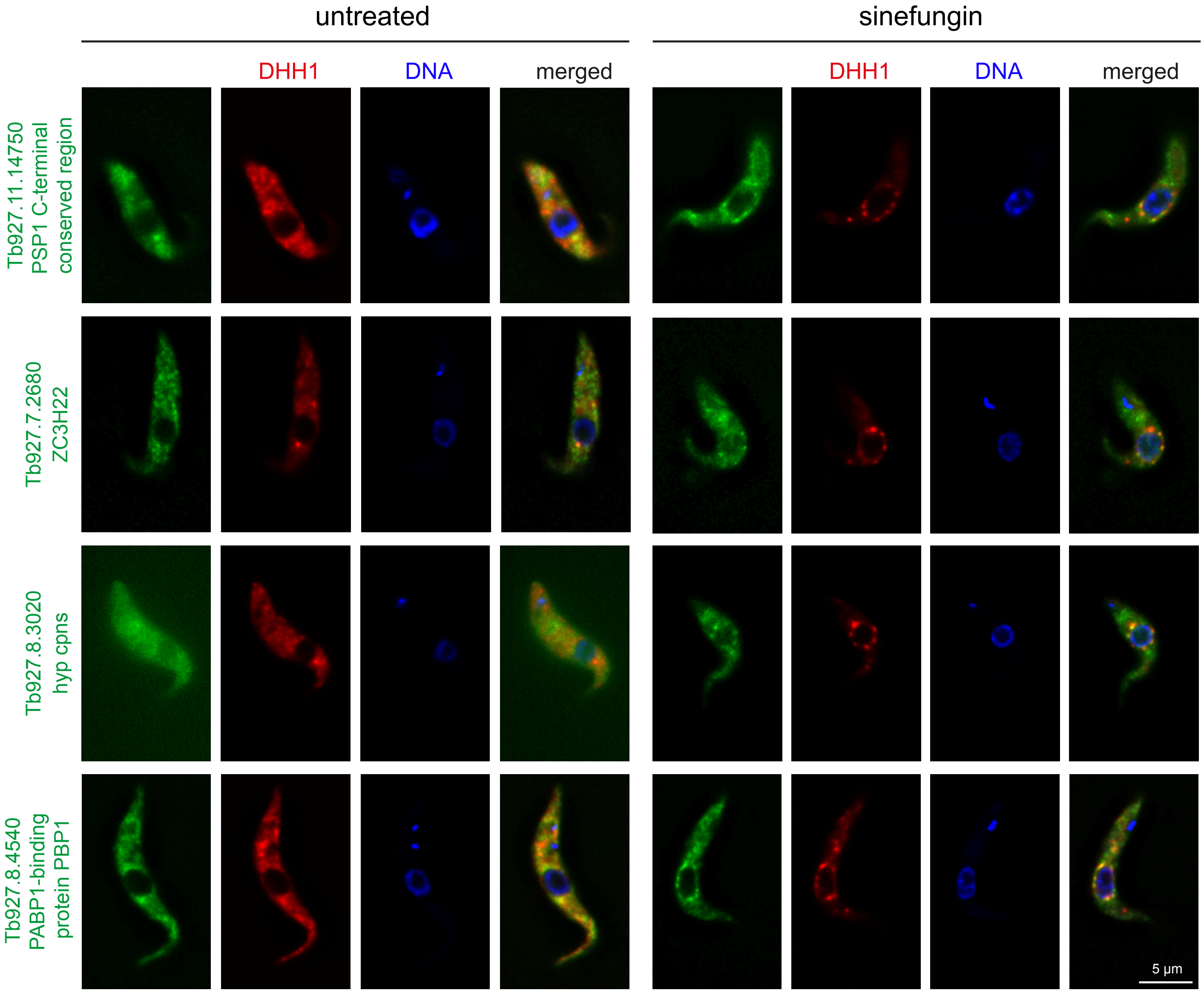

Supplementary Fig. 10: ESTN proteins that localise to NPGs
Representative microscopy images of one untreated and one sinefungin-treated cell are shown. DNA was stained with DAPI. For most images, single plane images of deconvolved z-stacks are shown. In some cases, z-stack projections of a subset of images are shown (for details see Supplementary Table 1A).

Supplementary Figure 11

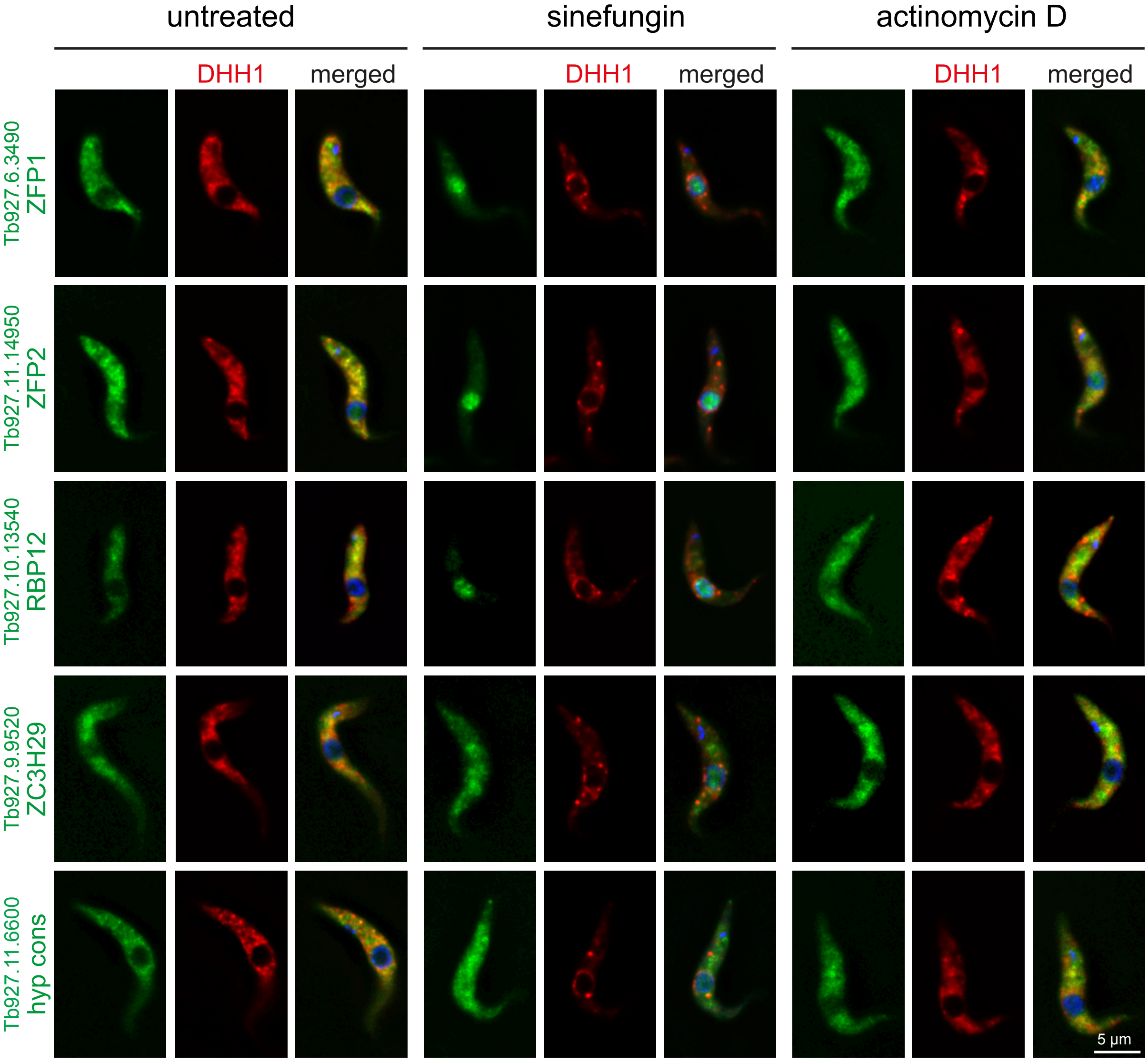

Supplementary Fig. 11: ESTN proteins that re-localise to the nucleus in response to sinefungin treatment.

Z-stack projections of one untreated, one sinefungin-treated and one actinomycin D-treated (10 µg/ml, 60 min) cell are shown for each protein. Note that actinomycin D treatment does not cause relocalisation of the proteins to the nucleus, evidence that it is the presence of unspliced mRNAs, rather than the absence of newly transcribed mRNAs that causes the relocalisation.

Supplementary Figure 12

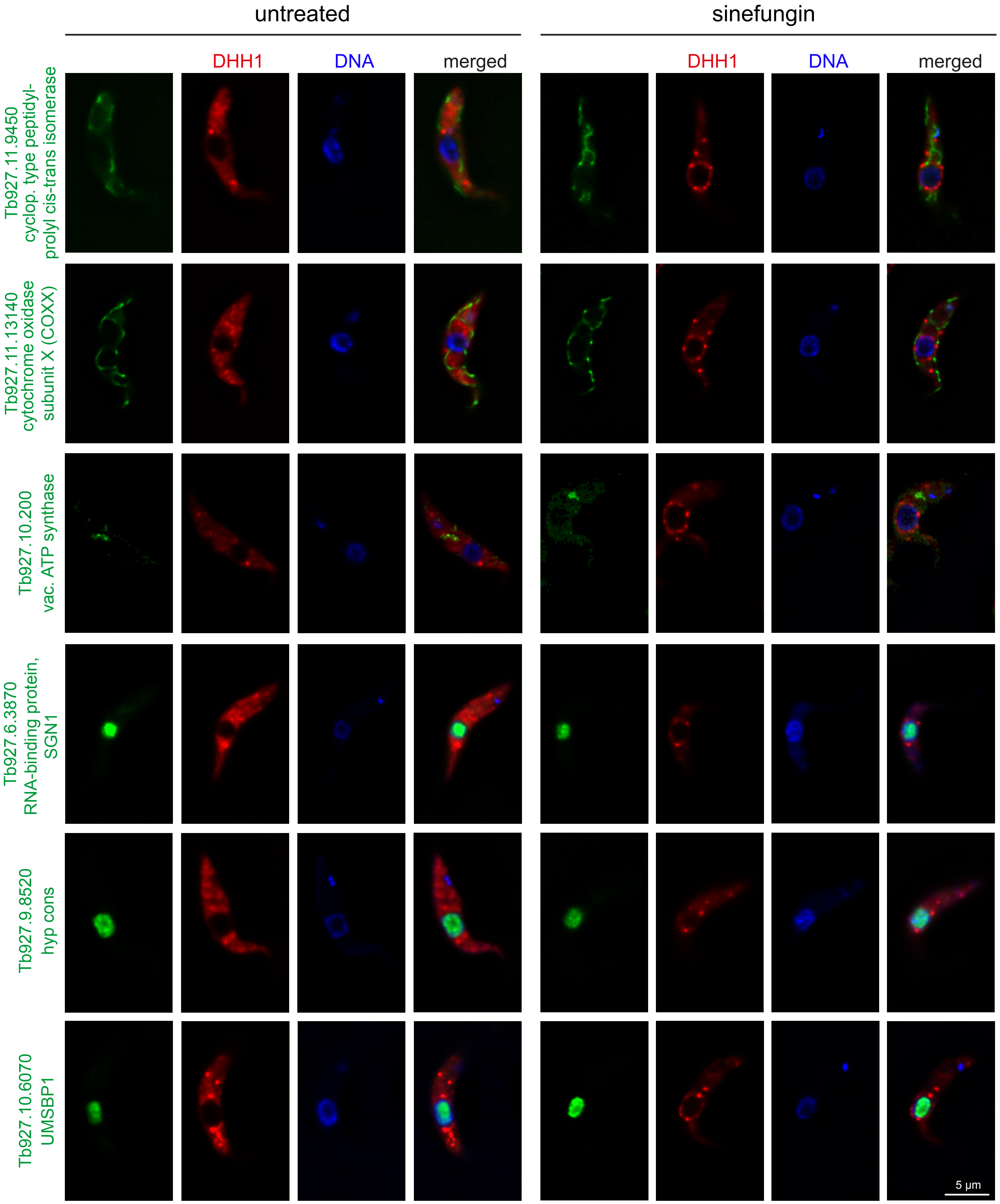

Supplementary Figure 12

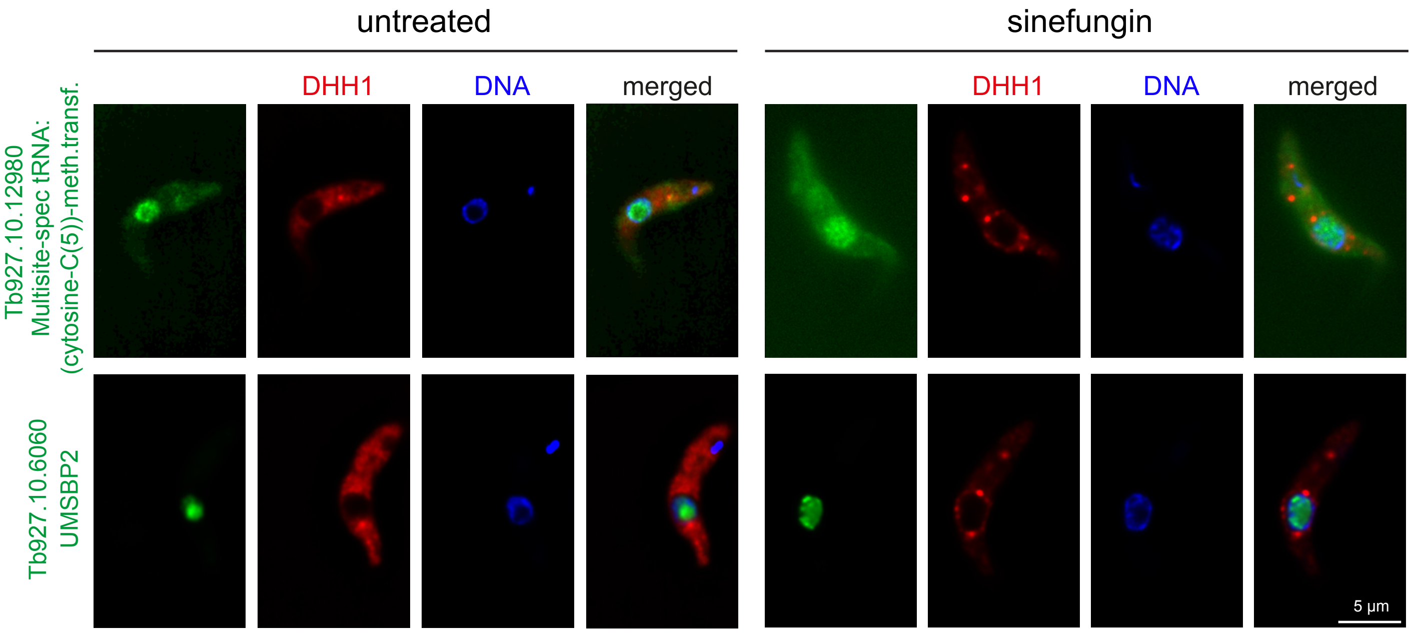

Supplementary Fig. 12: ESTN proteins that do not change their localisation at sinefungin treatment

Representative microscopy images of one untreated and one sinefungin-treated cell are shown. DNA was stained with DAPI. Images are either shown as single plane images of deconvolved Z-stacks or as Z-stack projections (for details see Supplementary Table 1A).

Supplementary Figure 13

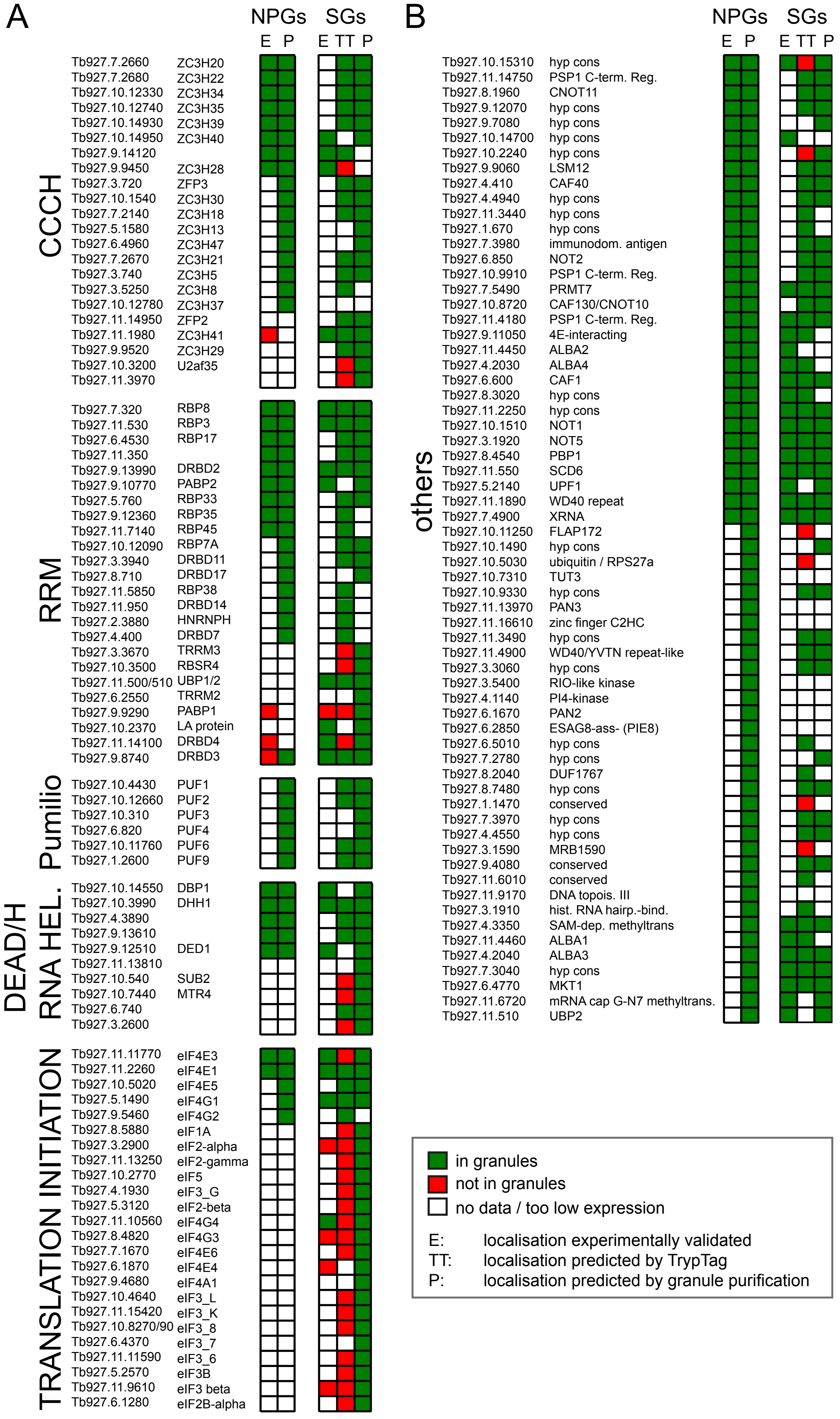

Supplementary Figure 13: NGPs resemble stress granules in composition

The 113 likely NPG proteins (experimentally validated=E or predicted=P) were grouped according to their RNA binding domain or function as CCCH, RRM, Pumilio, DEAD/H RNA helicases, translation initiation factors **(A)** or others **(B)**. For comparison, data from the stress granule proteome[1)] were added, with a colour-code to show positive (green) or negative (red) localisation to stress granules by localisation experiments (E) (citations in Supplementary Table 1), predicted from the purification (P) or predicted by the genome-wide localisation project TrypTag (TT) [2)]. The most reliable data are from experiments, as these also contained a stress granule marker protein and cells were starved for carbon sources by incubation in PBS. TrypTag images also show stress granules, as the cells were kept in amino acid free solution (vPBS) for several hours prior to imaging. The stress granule proteome was obtained by a size-based fractionation method and does contain many false-positives; these are proteins that form aggregates other than stress granules in response to starvation [1)].

The comparison between the proteomes of the two different granule types reveals remarkable similarities. The same six Pumilio proteins are predicted in both NPGs and SGs, and very similar sets of CCCH and RRM domain proteins. No data from experimental validations suggest that a certain protein is specific for either NPGs or SGs, with the exception of DRBD3 and DRBD4 that appear unique to SGs: however, both proteins are partially (DRBD3) or entirely (DRBD4) nuclear and granules at the nuclear periphery are therefore difficult to visualise. Of all 117 proteins predicted or validated in NPGs, 91 were shown either by localisation experiments of starved cells (E) or by TrypTag (TT) to localise also to stress granules and only for one protein with no experimental data, Tb927.10.2240, TrypTag data indicate no SG localisation; we have tagged this protein, but the expression level was too low to unequivocally confirm or exclude granule localisation.

Vice versa, within the groups of the CCCH, RRM, Pumilio and helicase proteins there are only 18 additional predicted SG proteins with no evidence for NPG localisation of which at least 9 are likely not in SGs as evidenced by experiments or TrypTag. Of particular interest is the group of translation initiation factors. Only five proteins, all isoforms of the eIF4F complex, are predicted in NPGs (eIF4E1,3,5 and eIF4G1,2) and these five have strong evidence for SG localisation too; in three cases this is shown by experimental validation in the other two cases by TrypTag. Many other translation initiation factors are predicted in SGs by the purification, but experimental validation or TrypTag prediction indicates no localisation to SGs for most of these proteins. Notably, the proteins that are currently considered the major components of the eIF4F complex, eIF4E4 and eIF4G3, [3)] are absent from both granule types. Thus, even though there are major differences between SGs and NPGs in localisation and sensitivity to transcriptional and translational inhibitors, their composition is very similar.

[1)] Fritz, M. et al. Novel insights into RNP granules by employing the trypanosome's microtubule skeleton as a molecular sieve. Nucleic Acids Research 43, 8013-8032 (2015).

[2)] Dean, S., Sunter, J. D. & Wheeler, R. J. TrypTag.org: A Trypanosome Genome-wide Protein Localisation Resource. Trends in Parasitology 33, 80-82 (2017).

[3)] Freire, E. R., Sturm, N. R., Campbell, D. A. & de Melo Neto, O. P. The Role of Cytoplasmic mRNA Cap-Binding Protein Complexes in Trypanosoma brucei and Other Trypanosomatids. Pathogens (Basel, Switzerland) 6 (2017).

Supplementary Figure 14

A

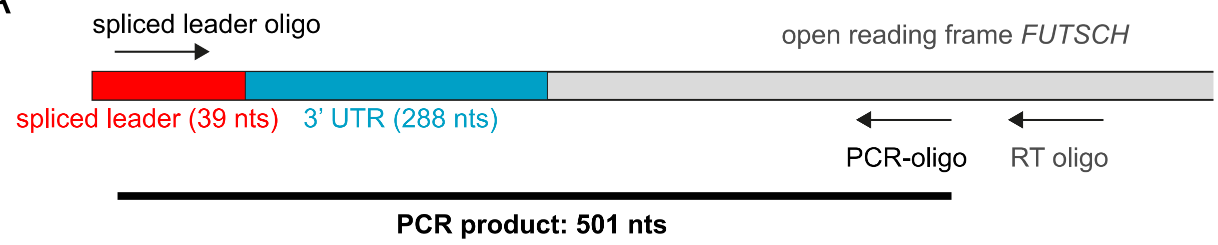

B

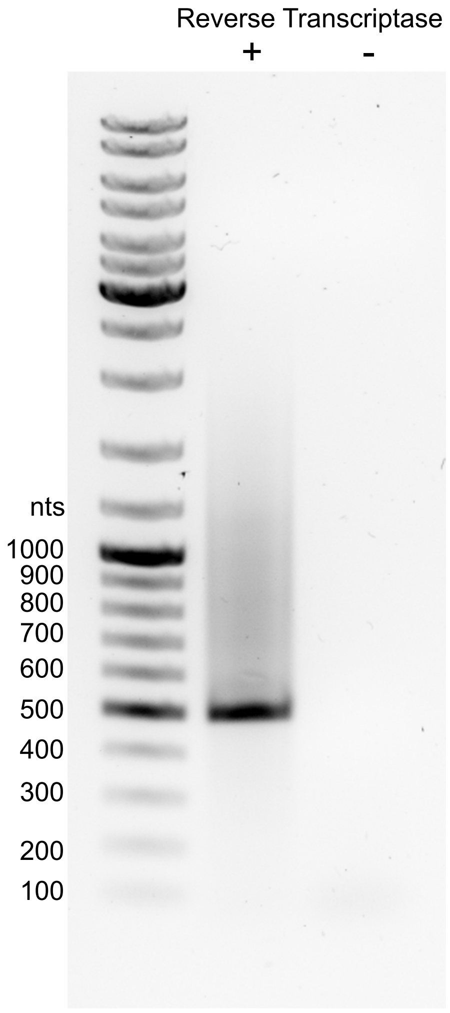

Supplementary Figure 14: RT-PCR to confirm presence of miniexon in FUTSCH mRNA.
A) The RT-PCR strategy is shown schematically (not to scale).
B) RT PCR: To reduce the complexity of the sample and allow RT-PCR detection of a a very low-abundant RNA, uncapped RNAs were removed by XRN1 digestion. For this, 45 µg total trypanosome RNA (from PCFs) were incubated for 1 hour at 37°C in the presence of 3 µl XRN1 (NEB), 10 µl NEB buffer 3 and 3 µl Ribolock (Thermofischer) in a total volume of 100 µl. The RNA was cleaned by phenol extraction and ethanol precipitation and resuspended in 10 µl RNAse-free water. 0.5 µl of these capped RNAs were used as templates in the reverse transcription reaction with Maxima_H_Minus Reverse Transcriptase (Fermentas) which was performed according to the protocol of the manufacturers. Reverse transcription was first done for 10 min at 25°C, followed by 30 min at 50°C. Then, 1 µl RNAse H (NEB) was added to each reaction and the samples were incubated for an additional 60 min at 37°C, before the reaction was stopped (5 min, 85°C). 2 µl of the reverse transcription reaction was used in the subsequent hot start PCR with Expand high fidelity DNA polymerase (Roche). The PCR was done for 40 cycles with 55°C annealing temperature and 45’’ elongation time. For the negative control, the reverse transcription reaction was performed in the absence of reverse transcriptase.
5‘-3’ sequences of the oligos are:
RT oligo: GCAAGGCAATCGTTGTCTC
PCR oligo: GTACAACTTTTCGTATCAC
spliced leader oligo: GAACAGTTTCTGTACTATAT
